# Co/Ni-polyoxotungstate photocatalysts as precursor materials for electrocatalytic water oxidation†

**DOI:** 10.1039/d0ra10792a

**Published:** 2021-03-18

**Authors:** Robin Güttinger, Giann Wiprächtiger, Olivier Blacque, Greta R. Patzke

**Affiliations:** Department of Chemistry, University of Zurich Winterthurerstrasse 190 CH-8057 Zurich Switzerland greta.patzke@chem.uzh.ch http://www.patzke.ch

## Abstract

An open-core cobalt polyoxometalate (POM) [(A-α-SiW_9_O_34_)Co_4_(OH)_3_(CH_3_COO)_3_]^8−^Co(1) and its isostructural Co/Ni-analogue [(A-α-SiW_9_O_34_)Co_1.5_Ni_2.5_(OH)_3_(CH_3_COO)_3_]^8−^CoNi(2) were synthesized and investigated for their photocatalytic and electrocatalytic performance. Co(1) shows high photocatalytic O_2_ yields, which are competitive with leading POM water oxidation catalysts (WOCs). Furthermore, Co(1) and CoNi(2) were employed as well-defined precursors for heterogeneous WOCs. Annealing at various temperatures afforded amorphous and crystalline CoWO_4_- and Co_1.5_Ni_2.5_WO_4_-related nanoparticles. CoWO_4_-related particles formed at 300 °C showed substantial electrocatalytic improvements and were superior to reference materials obtained from co-precipitation/annealing routes. Interestingly, no synergistic interactions between cobalt and nickel centers were observed for the mixed-metal POM precursor and the resulting tungstate catalysts. This stands in sharp contrast to a wide range of studies on various heterogeneous catalyst types which were notably improved through Co/Ni substitution. The results clearly demonstrate that readily accessible POMs are promising precursors for the convenient and low-temperature synthesis of amorphous heterogeneous water oxidation catalysts with enhanced performance compared to conventional approaches. This paves the way to tailoring polyoxometalates as molecular precursors with tuneable transition metal cores for high performance heterogeneous electrocatalysts. Our results furthermore illustrate the key influence of the synthetic history on the performance of oxide catalysts and highlight the dependence of synergistic metal interactions on the structural environment.

## Abstract 2

Open-core polyoxometalates are powerful precursors with pre-organized metal centers for the tunable synthesis of cobalt tungstate-based water oxidation electrocatalysts.

## Graphical abstract

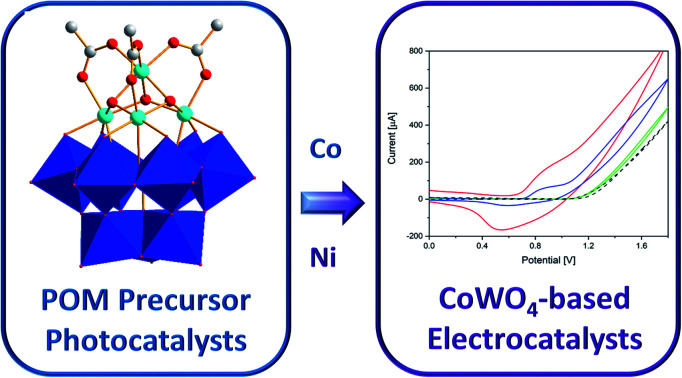

## 1. Introduction

Sunlight-driven splitting of water into hydrogen and oxygen, also known as artificial photosynthesis, is among the most direct and elegant one-step concepts for renewable energy sources.^1^ However, the development of efficient and noble-metal free water oxidation catalysts (WOCs) for this complex four electron transfer process is still a crucial bottleneck of artificial photosynthesis.^2^ A variety of approaches to WOC design have been reported, and in many of them the cuboidal {CaMn_4_O_5_} core of nature's photosystem II is a central motif.^3^ Recently, several transition metal WOCs with cubane-related cores have been reported.^4–10^

Polyoxometalates (POMs) are promising WOC candidates, because they combine robustness and structural versatility with the capability of undergoing rapid, reversible, and stepwise multi-electron transfer reactions, and we also refer to key review articles here.^11–16^ Among the attractive and low cost 3d transition metal water splitting catalysts, cobalt-based homogeneous catalysts and POMs keep attracting intense attention.^17–22^ Specifically, Co-POMs have recently been applied as co-catalysts on photoanodes or for enhanced performance in composite systems.^23–28^.

The coordination of multiple metal centers between two or more lacunary POM units has proven a powerful and quite flexible catalytic motif, such as in the OEC-related [Mn^III^_3_Mn^IV^O_3_(CH_3_COO)_3_(SiW_9_O_34_)]^6−^ with a mixed-valent {Mn_4_} core.^29^ Although many POMs have wide operational stability windows, they can undergo leaching of heteroatoms/transition metals to form active nanoscale oxide catalysts, especially during electrochemical water oxidation.^30,31^ This gives rise to ongoing and challenging speciation studies.^32^

Recently, the performance of an amorphous sandwich-type Co-based POM WOC was first enhanced by annealing at 400 °C to form CoWO_4_ nanoparticles, which outperformed analogous electrocatalysts obtained *via* precipitation routes.^33^ Along these lines, mixed-metal manganese cubane^34^ and Ni–Zn cubane-like^35^ precursors were further applied to enhance the performance of oxide WOCs. These findings show that synthetic methods and underlying mechanisms^36^ are an important step in WOC optimization,^37–39^ and that a certain extent of pre-organization of the metal centers in the precursor is beneficial for higher catalytic activity.^40^ To this end, we systematically explored synthesis-activity relationships with a series of studies on spinel-type Co_3_O_4_ catalysts, starting with *in situ* PXRD monitoring of temperature-dependent hydrothermal Co_3_O_4_ formation mechanisms.^41^ Next, we revealed the preparation-dependent properties of Co_3_O_4_ WOCs in different test assays,^42^ and studies of their microwave-assisted synthesis further confirmed the crucial role of synthetic pathways for the catalytic performance.^43^

POM as oxide precursors have enabled the efficient synthesis of CoO_*x*_ electrocatalysts^44^ or of ultrafine transition metal-clusters,^45^ as well as inspirational studies on transformation pathways of pre-organized metal centers into structural features of the resulting multinary heterogeneous catalysts.^46,47^ Recent trends further employed POMs as versatile metal sources for carbide-, phosphide- and sulphide-based water splitting electrocatalysts.^48–50^ All in all, the complexity of such precursor-properties relations remains to be fully explored and understood for efficient WOC design.

Another crucial principle in the optimization of transition metal catalysts are synergistic effects between mixed metal centers, such as the widely studied Ni/Fe interactions in WOCs.^51–53^ In comparison, Co/Ni-interactions in oxygen evolution and other catalysts are far more diverse and controversial. Mixed heterogeneous Co/Ni-oxide electrocatalysts have been studied for several decades^54^ and were frequently reported to be favourable over binary systems.^55–57^ However, later studies on mixed Co/Ni-hydroxides pointed to either productive^58^ or adverse^59^ effects, or to no significant interactions in the case of oxides at all.^60^ Although Co/Ni synergisms were recently observed for sulphide^61^ and phosphide^62^ water splitting electrocatalysts, their understanding is still empirical to a large extent and modelling studies are now being undertaken.^62^ Even less is known about the effect of Co/Ni-substitution on molecular WOCs and other catalysts. In our own work, for example, we have observed drastic contrasts between the notable improvement of solid CoNCN WOCs through Ni-doping^63^*vs.* detrimental effects on molecular {Co(ii)_4_O_4_} cubane WOCs.^64^ Similar adverse Co/Ni-interactions have been reported for other molecular systems.^65^ Generally, a wide range of further studies is now needed to explore the role of materials type, preparative method and test conditions (photo- *vs.* electrochemistry) in the performance of Co/Ni-based catalysts. These widely unresolved questions concerning the prediction and explanation of Co/Ni-interactions inspired us herein to first investigate molecular photocatalyst performance with respect to Ni introduction, followed by its effect on the solid electrocatalysts obtained from such molecular precursors.

To this end, we selected Co/Ni-POMs as attractive and comprehensive models to investigate (a) Co/Ni interactions in molecular WOCs *vs.* (b) those in oxide-based catalysts, while (c) exploring the benefits of POM precursors for oxide WOCs.

To this end, we targeted CoWO_4_ with favourable 500–650 nm light absorption properties that was also reported as an effective and noble metal-free WOC at low overpotential.^66^ Furthermore, CoWO_4_ performance was found to depend on crystallinity^67^ with amorphous CoWO_4_ being superior to its crystalline form, along the lines of self-repairing CoPi films.^68–70^ However, little is still known about the electrocatalytic performance and other applications of mixed Co/Ni-tungsten oxides.^71,72^ Binary CoWO_4_ and NiWO_4_, for example, display better electrochemical performance in water oxidation than NiCo_2_O_4_ spinels.^72^ While mixed (Co, Ni)WO_4_ materials are attractive for supercapacitor development,^73,74^ no synergistic benefits were reported for their use in photocatalytic methylene blue degradation.^75^

We therefore newly investigated the influence of Ni-doping on the performance of CoWO_4_-related water oxidation catalysts, which were obtained from crystallographically well-defined, bio-inspired M_4_-POMs with an exposed metal core architecture. While Ni-containing POMs were reported to be stable and active for water oxidation,^76,77^ their potential as mixed metal precursors for heterogeneous WOCs still needs to be explored. To this end, we synthesized and characterized [(A-α-SiW_9_O_34_)Co_4_(OH)_3_(CH_3_COO)_3_]^8−^Co(1) together with its isostructural analogue [(A-α-SiW_9_O_34_)Co_1.5_Ni_2.5_(OH)_3_(CH_3_COO)_3_]^8−^CoNi(2).^78^ First, both POMs were compared with respect to their respective photo- and electrocatalytic water oxidation activity. Moreover, they were used as annealing precursors to form CoWO_4_- and mixed (Co,Ni)WO_4_-related electrocatalysts, and Co(1) was found to be an efficient precursor at temperatures as low as 300 °C.

## 2. Experimental

### 2.1 Materials

All chemicals were used as purchased without purification. The lacunary precursor Na_10_[A-α-SiW_9_O_34_]·19H_2_O was synthesized as previously described.^79^

### 2.2 Physical methods

Attenuated total reflectance Fourier-transform (ATR-FT-IR) spectra were recorded on a Bruker Vertex 70 spectrometer equipped with a Platinum ATR accessory containing a diamond crystal. UV/Vis spectra were recorded on a Lambda 650 S PerkinElmer UV-visible spectrometer in the range of 200–800 nm using a Quartz SUPRASIL precision cell (10 mm). Raman spectroscopy was recorded with a Renishaw inVia Qontor confocal Raman microscope equipped with a diode laser (785 nm). Thermogravimetric analyses were performed on a Netzsch STA 449C between 24 and 600 °C with a heating rate of 10 K min^−1^ in N_2_ atmosphere and Al_2_O_3_ crucible. PXRD patterns were recorded on a STOE STADI P diffractometer (transmission mode, Ge monochromator) with Cu or Mo radiation. XPS analysis was performed using a PhI 5000 VersaProbe spectrometer (ULVAC-PHI, Inc.) equipped with a 180° spherical capacitor energy analyzer and a multi-channel detection system with 16 channels. Spectra were acquired at a base pressure of 5 × 10^−8^ Pa using a focused scanning monochromatic Al-K_α_ source (1486.6 eV) with a spot size of 200 μm and 50 W. The instrument was run in the FAT analyzer mode with electrons emitted at 45° to the surface normal. Pass energy used for survey scans was 187.85 eV and 46.95 eV for detail spectra. Charge neutralisation utilizing both a cool cathode electron flood source (1.2 eV) and very low energy Ar^+^ ions (10 eV) was applied throughout the analysis.

*Visible-light-driven water oxidation* was first monitored in solution using an oxygen sensor (OX-N) Clark electrode from Unisense. Constant temperature was maintained with a mineral insulated thermosensor (2 mm tip diameter, TP2000, Unisense). Second, O_2_ evolution was measured in the headspace of the vial using an Agilent Technologies 7820A gas chromatograph with helium as the carrier gas and a 3 m × 2 mm packed molecular sieve 13 × 80–100 column to separate O_2_ and N_2_. The oven was operated isothermally at 100 °C. The analysis of the headspace was performed by taking 100 μL samples with a Hamilton (1825 RN) gas-tight microliter syringe. Gases were detected using a thermal conductivity detector (Varian) operated at 200 °C.

*Cyclic voltammetry (CV)* measurements were performed on a Metrohm 797 VA Computrace instrument with a platinum electrode (Metrohm AG, 2 mm diameter) as a working electrode, Ag/AgCl reference electrode (sat. KCl, 0.197 V *vs.* NHE) and platinum plate (Metrohm AG) counter electrode. Prior to all measurements, solutions were deaerated with Ar for 15 min. The platinum working electrode was polished between runs with alumina slurry, thoroughly rinsed with water and dried under ambient conditions. The platinum plate was washed in a nitric acid/hydrogen peroxide (1 : 1) solution for 5 min and dried with N_2_. The working electrodes were produced by dispersing 5 mg of the sample in 100 μL of H_2_O, applying 40 μL of this dispersion on 1 cm^2^ fluorine doped tin oxide (FTO), and drying the electrodes at 80 °C for 30 min before covering with 10 μL Nafion 1% solution.

### 2.3 K_5_Na_3_[(A-α-SiW_9_O_34_)Co_4_(OH)_3_(CH_3_COO)_3_]·15H_2_O (1)^78^

Co(CH_3_COO)_2_·4H_2_O (0.712 g, 2.86 mmol) was dissolved in an aqueous solution of potassium acetate (0.5 M, 16 mL), adjusted to pH 8 with HCl and stirred for 15 min. Na_10_[A-α-SiW_9_O_34_]·19H_2_O (1.977 g, 0.7 mmol) was added and stirred for 45 min at 40 °C. The mixture was then cooled to room temperature and placed in the fridge for 10 min. The purple suspension was centrifuged, filtered and left at room temperature for slow evaporation. After three weeks purple crystals were collected and analysed by FT-IR and Raman spectroscopy, powder X-ray diffraction, ICP-MS, EDX and ESI-MS. (yield 0.34 g, 15% based on tungsten). FT-IR: *ν̃* = 1598 (m), 1552 (m), 1415 (m), 1350 (w), 979 (w), 931 (m), 883 (s), 792 (s), 665 (s), 514 (s), 451 cm^−1^(m). ESI-MS: 879.3938 [M-(CH_3_COO) + 5H^+^]^3−^.

### 2.4 K_5_Na_3_[(A-α-SiW_9_O_34_)Co_1.5_Ni_2.5_(OH)_3_(CH_3_COO)_3_]·16H_2_O (2)

Co(CH_3_COO)_2_·4H_2_O (0.352 g, 1.41 mmol) and Ni(CH_3_COO)_2_·4H_2_O (0.366 g, 1.47 mmol) were dissolved in an aqueous solution of potassium acetate (1 M, 16 mL), adjusted to pH 8 with HCl and stirred for 15 min. Na_10_[A-α-SiW_9_O_34_]·19H_2_O (1.513 g, 0.53 mmol) was added and stirred for 45 min at 40 °C. The mixture was then cooled to room temperature and placed in the fridge for 10 min. The purple suspension was centrifuged, filtered and left at room temperature for slow evaporation. After two weeks light purple crystals were collected and analysed by FT-IR and Raman spectroscopy, powder X-ray diffraction, ICP-MS, EDX and ESI-MS (yield 0.23 g, 13% based on tungsten). FT-IR: *ν̃* = 1606 (m), 1556 (m), 1421 (m), 1350 (w), 979 (w), 941 (m), 883 (s), 802 (s), 665 (s), 514 (s), 451 cm^−1^ (m). ESI-MS: 876.7113 [M-(CH_3_COO) + 5H^+^]^3−^.

#### 2.4.1 CoW200

K_5_Na_3_[(A-α-SiW_9_O_34_)Co_4_(OH)_3_(CH_3_COO)_3_]·15H_2_O (0.043 g) was added into a crucible and placed in a furnace which was heated to 200 °C with a ramping temperature of 5 °C min^−1^ and annealed for 1 h to yield 0.04 g. After cooling down to room temperature, the violet compound was analysed with FT-IR and Raman spectroscopy as well as PXRD. FT-IR: *ν̃* = 1560 (m), 1404 (m), 1338 (w), 1116 (w), 987 (w), 939 (m), 865 (s), 779 (s), 661 (s), 524 cm^−1^ (m).

#### 2.4.2 CoW300

K_5_Na_3_[(A-α-SiW_9_O_34_)Co_4_(OH)_3_(CH_3_COO)_3_]·15H_2_O (0.087 g) was added into a crucible and placed in a furnace which was heated to 300 °C with a ramping temperature of 5 °C min^−1^ and annealed for 1 h to yield 0.08 g. After cooling down to room temperature the black compound was analysed by FT-IR and Raman spectroscopy as well as PXRD. FT-IR: *ν̃* = 1564 (w), 1404 (w), 1340 (w), 1128 (w), 939 (m), 852 (s), 771 (s), 702 (s), 538 cm^−1^ (m).

#### 2.4.3 CoW400

K_5_Na_3_[(A-α-SiW_9_O_34_)Co_4_(OH)_3_(CH_3_COO)_3_]·15H_2_O (0.305 g) was added into a crucible and placed in a furnace which was heated to 400 °C with a ramping temperature of 5 °C min^−1^ and annealed for 1 h to yield 0.27 g. After cooling down to room temperature the grey compound was analysed by FT-IR and Raman spectroscopy as well as PXRD. FT-IR: *ν̃* = 1128 (m), 929 (w), 823 (s), 790 (s), 599 (s), 518 (s), 460 cm^−1^ (m).

#### 2.4.4 CoW500

K_5_Na_3_[(A-α-SiW_9_O_34_)Co_4_(OH)_3_(CH_3_COO)_3_]·15H_2_O (0.09 g) was added into a crucible and placed in a furnace which was heated to 500 °C with a ramping rate of 5 °C min^−1^ and annealed for 1 h to yield 0.08 g. After cooling down to room temperature the dark blue compound was analysed with FT-IR and Raman spectroscopy as well as PXRD. FT-IR: *ν̃* = 1114 (m), 931 (w), 819 (s), 599 (s), 516 (s), 462 cm^−1^ (s).

#### 2.4.5 CoNi200

K_5_Na_3_[(A-α-SiW_9_O_34_)Co_1.5_Ni_2.5_(OH)_3_(CH_3_COO)_3_]·16H_2_O (0.021 g) was added into a crucible and placed in a furnace which was heated to 200 °C with a ramping rate of 5 °C min^−1^ and annealed for 1 h to yield 0.02 g. After cooling down to room temperature the purple compound was analysed with FT-IR and Raman spectroscopy as well as PXRD. FT-IR: *ν̃* = 1562 (m), 1407 (m), 1344 (w), 1122 (w), 981 (w), 933 (m), 858 (s), 798 (s), 671 (s), 520 cm^−1^ (m).

#### 2.4.6 CoNi300

K_5_Na_3_[(A-α-SiW_9_O_34_)Co_1.5_Ni_2.5_(OH)_3_(CH_3_COO)_3_]·16H_2_O (0.087 g) was added into a crucible and placed in a furnace which was heated to 300 °C with a ramping rate of 5 °C min^−1^ and annealed for 1 h to yield 0.08 g. After cooling down to room temperature the black compound was analysed with FT-IR and Raman spectroscopy as well as PXRD. FT-IR: *ν̃* = 1569 (w), 1404 (w), 1128 (w), 933 (m), 838 (s), 788 (s), 711 (s), 540 cm^−1^ (m).

#### 2.4.7 CoNi400

K_5_Na_3_[(A-α-SiW_9_O_34_)Co_1.5_Ni_2.5_(OH)_3_(CH_3_COO)_3_]·16H_2_O (0.114 g) was added into a crucible and placed in a furnace which was heated to 400 °C with a ramping rate of 5 °C min^−1^ and annealed for 1 h to yield 0.10 g. After cooling down to room temperature the grey compound was analysed with FT-IR and Raman spectroscopy as well as PXRD. FT-IR: *ν̃* = 1107 (w), 941 (m), 858 (s), 813 (s), 773 (s), 725 cm^−1^ (s).

#### 2.4.8 CoNi500

K_5_Na_3_[(A-α-SiW_9_O_34_)Co_1.5_Ni_2.5_(OH)_3_(CH_3_COO)_3_]·16H_2_O (0.057 g) was added into a crucible and placed in a furnace which was heated to 500 °C with a ramping rate of 5 °C min^−1^ and annealed for 1 h to yield 0.05 g. After cooling down to room temperature the dark green compound was analysed with FT-IR and Raman spectroscopy as well as PXRD. FT-IR: *ν̃* = 1116 (m), 933 (w), 821 (s), 605 (s), 509 (s), 466 cm^−1^ (m).

#### 2.4.9 Reference CoWO_4_

An aqueous solution of Na_2_WO_4_ (0.1 M, 10 mL) was added dropwise to a Co(NO_3_)_2_ solution (0.1 M, 10 mL) under vigorous stirring. The precipitate was rinsed with water after centrifugation and dried overnight in the oven (40 °C). The collected precipitate was added into a crucible and placed in a furnace which was heated to 300 °C with a ramping rate of 5 °C min^−1^ and annealed for 1 h.^66^

## 3. Results and discussion

### 3.1 Synthesis and analytical characterization

[(A-α-SiW_9_O_34_)Co_4_(OH)_3_(CH_3_COO)_3_]^8−^Co(1) was synthesized by mixing stoichiometric amounts of the precursor^80^ Na_10_[A-α-SiW_9_O_34_] and cobalt acetate in potassium acetate (0.5 M, pH 8) solution with moderate heating. After cooling to room temperature, the mixture was filtered and any insoluble residue was removed, whereupon crystals were obtained after slow evaporation.

FT-IR analysis shows characteristic bands for the bidentate bridging acetate ligands in the range of 1650 to 1400 cm^−1^. Additional bands related to the Keggin structure appear around 934 (*ν*_as_(W–O_d_)), 883 (*ν*_as_(W–O_b_)) and 740 cm^−1^ (*ν*_as_(W–O_c_)) (Fig. S1†).^81^ Raman spectra show representative peaks at 959 cm^−1^ (*ν*_as_(W–O_d_)), 939 cm^−1^ (*ν*_as_(W–O_b_–W)) and 891 cm^−1^ (*ν*_as_(W–O_b_–W)) of Keggin-type POMs (see below, Fig. 3).^80,82^ Further analysis with powder X-ray diffraction (PXRD) of Co(1) (Fig. S13†) showed crystalline purity when compared to the calculated pattern (CCDC-619251).

The all-cobalt POM Co(1) was further mixed with nickel acetate in a stoichiometric 1 : 1 ratio under slightly changed reaction conditions. The filtration process after the synthesis had to be extended, but phase pure crystals were obtained after slow evaporation and yielded [(A-α-SiW_9_O_34_)Co_1.5_Ni_2.5_(OH)_3_(CH_3_COO)_3_]^8−^CoNi(2). FT-IR analysis confirms the presence of the bridging acetate ligands as well as the characteristic bands of the Keggin-type POM at around 941 (*ν*_as_(W–O_d_)), 883 (*ν*_as_(W–O_b_)) and 748 cm^−1^ (*ν*_as_(W–O_c_)) (Fig. S2†).^81^ The PXRD pattern confirmed phase purity of CoNi(2) and its isostructural relation to Co(1), and only small peak shifts of the peaks towards higher angles are visible which corresponds to a smaller unit cell, as expected (Fig. S16†). Raman spectra show the same representative peaks as observed for Co(1) (see below, Fig. 3).

**Fig. 1:**
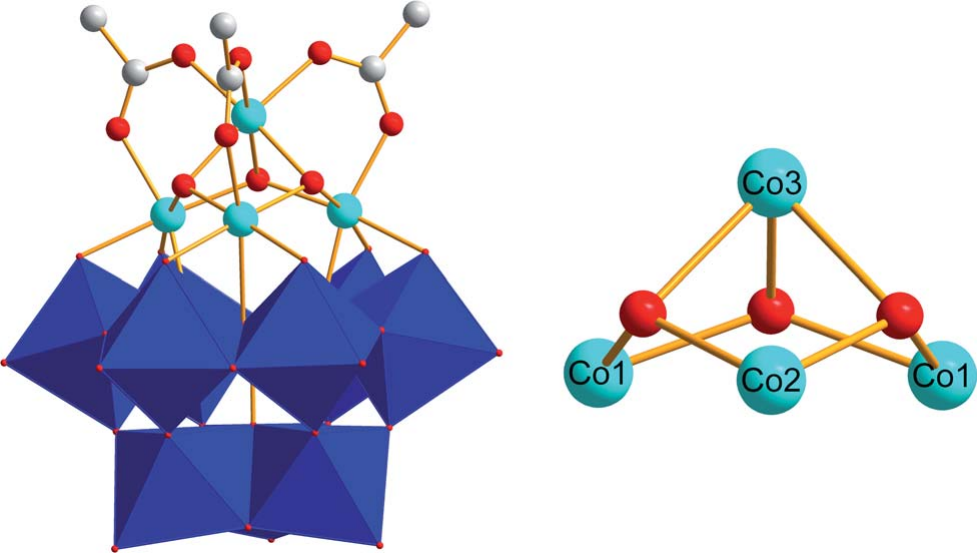
Polyhedral and ball-and-stick representation of the [(A-α-SiW_9_O_34_)Co_4_(OH)_3_(CH_3_COO)_3_]^8−^ polyanion Co(1) (blue octahedra: {WO_6_}; light blue spheres: Co; white spheres: C; red spheres: O; image derived from CCDC-619251).^71^

UV-Vis monitoring of Co(1) in borate buffer (0.1 M, pH 8) during 24 h showed no significant changes in the spectra. This indicates that Co(1) is stable and does not leach any Co^2+^ ions into the solution under these operational conditions (Fig. S6†). According to previous studies,^78^ the tetracobalt core is stabilized by an all-inorganic tungstosilicate, as well as bridged by three μ_2_-acetate ligands (Fig. 1). All Co^II^ centers of the {Co^II^_4_O_3_} core are in an octahedral environment and the whole unit displays *C*_S_ symmetry, with a mirror plane through the Co_3_, Co_2_ and Si atoms. Three Co^II^ centers are connected to the lacunary side of the [α-SiW_9_O_34_]^10−^ POM. The Co–Co distances fall in the range of 2.978(1)–3.711(2) Å and the Co–O distances range from 2.043(0) to 2.117(7) Å, respectively.^78^ We confirmed the presence of these structural features in Co(1) with single crystal X-ray diffraction analyses giving rise to analogous values (data not shown). The tetracobalt core displays features related to the natural OEC with Mn–Mn distances in the range of 2.8–3.3 Å,^3^ and the acetate-bridged cobalt centers relate it to previously reported {Co^II^_4_O_4_} cubanes.^6,83,84^

In addition, mixed nickel/cobalt acetate precursors yielded the iso-structural CoNi(2) with a Co : Ni ratio around 1.5 : 2.5. This ratio was confirmed with EDX, ICP-MS as well as with XPS measurements (Fig. S20, Tables S3, S11 and S12†). The measured Co/Ni : W as well as Co/Ni : Si ratios correspond to the respective calculated ratios of 4 : 9 and 4 : 1 (Tables S11 and S12†). Further HR-ESI-MS analyses showed slightly different masses for the corresponding [M-(CH_3_COO) + 5H^+^]^3−^ fragment which correspond to the isotopic distributions of Co and Ni (Fig. S29–S32†). Both POMs display good agreement between experimental PXRD patterns and the respective calculated data (Fig. S13, S15, Tables S1 and S2†). The PXRD pattern of CoNi(2) showed a slight shift of the peaks compared to the calculated reference pattern of Co(1) (CCDC-619251). Further comparison with the calculated PXRD pattern (Fig. S14†) of the lacunary Ni-analogue [(A-α-SiW_9_O_34_)Ni_4_(CH_3_COO)_3_]^5–^ that crystallizes in a different space group^85^ (*P*3̄1*c* other than *P*2_1_/*m* for Co(1)^78^) clearly showed that the phase pure CoNi(2) sample is isostructural with Co(1), which was also confirmed by Rietveld refinement results (Fig. S15†).

**Fig. 2:**
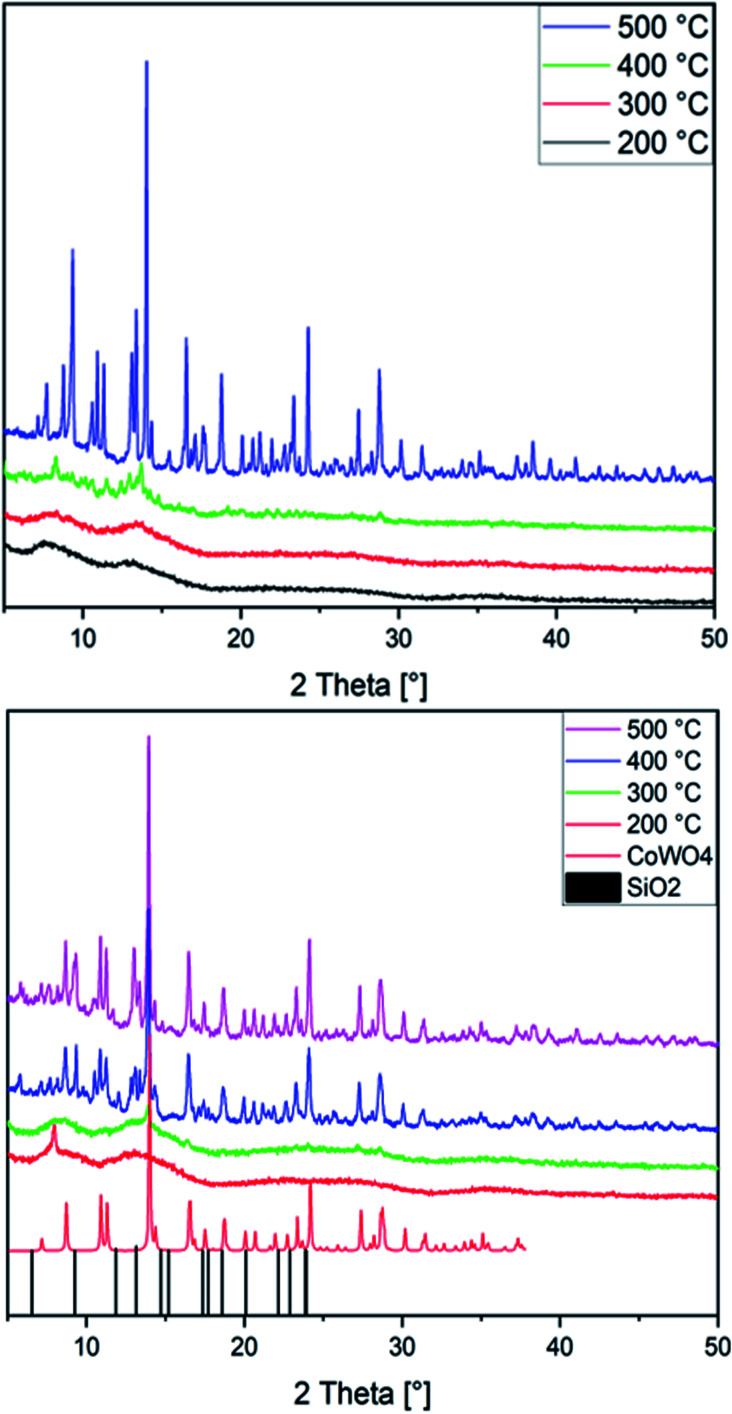
PXRD patterns of the CoNi*X*00 (top) and CoW*X*00 (bottom) series annealed at 200 °C (black) up to 500 °C (purple; CoWO_4_: CCDC-619251, SiO_2_: PDF No. 12-0711).

CoWO_4_ nanoparticles keep attracting intense interest as target for synthetic studies, *e.g. via* precipitation,^86,87^ hydrothermal^88,89^ or spray pyrolysis routes.^90^ Here, we newly used both Co(1) and CoNi(2) as precursors for annealing in air at temperatures ranging from 200 to 500 °C. With a ramping rate of 5 °C min^−1^ and an annealing time of 1 h, amorphous and crystalline nanoparticles were formed. PXRD patterns show the presence of an amorphous material up to 300 °C, while at temperatures of 400 °C and above a crystalline material emerges from both precursor types (Fig. 2). The majority of the peaks in patterns recorded with MoK_α_ radiation can be assigned to monoclinic CoWO_4_ (PDF 01-072-0479) and its Ni-doped analogue, in line with previous studies.^86,89,90^ While a preceding study on the use of sandwich-type [Co_4_(H_2_O)_2_(PW_9_O_34_)_2_]^10−^ POMs for cobalt tungstate catalysts reported on the formation of Na_2_W_2_O_7_ as a secondary phase at annealing temperatures of 500 °C,^33^ we did not find any indication for major W- or Co-based side products. Extensive database search provided SiO_2_ (PDF No. 12-0711) as the closest match to account for the minority phases observed here.

Given that SiO_2_ is widely known as a catalyst support material rather than as active phase, we further considered cobalt tungstosilicates a more reasonable precursor choice than tungstophosphates. The latter may eventually give rise to highly catalytically active cobalt phosphate-related side products. Indeed, phosphorus peaks had been shown in the EDS spectra of the most active amorphous cobalt tungstate catalyst obtained from [Co_4_(H_2_O)_2_(PW_9_O_34_)_2_]^10−^ at 400 °C in the above-mentioned study, but no further discussion of the influence of P heteroatoms on the structure or catalytic performance was provided.^33^

**Fig. 3:**
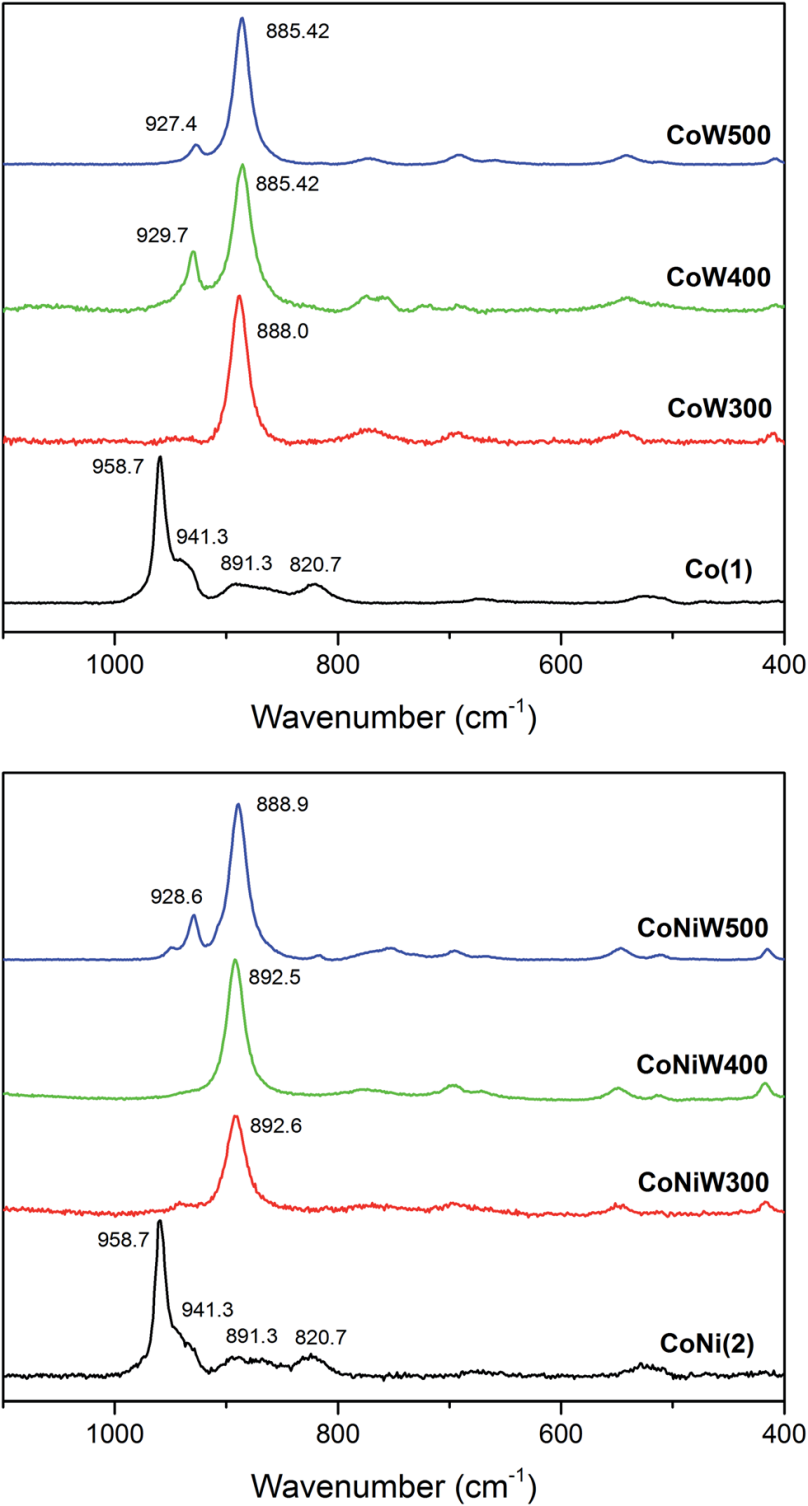
Raman spectra of Co(1) together with the CoW300/400/500 series (top; black/red/green/blue) and of compound CoNi(2) with the CoNiW300/400/500 series (bottom; black/red/green/blue).

The Raman spectra of crystalline CoW300/400/500 are in good agreement with the reported pattern of CoWO_4_ (Fig. 3).^91^ The most intense band located at 885 cm^−1^ corresponds to the stretching W–O vibration and is shifted to higher frequencies upon mixing with Ni. The small band around 929 cm^−1^ can be attributed to the symmetric stretching mode of the terminal (W=O) bond.^66,92^ The amorphous CoW300 and CoNi300/400 samples show a small blue shift of the main W–O stretching vibration compared to the crystalline samples. This is a sign of compressive stress, indicating that the respective Co–Co and Co–Ni distances are smaller compared to the crystalline samples.^92^ The weak peak around 500 cm^−1^ can be assigned to the minority phase related to SiO_2_.^93^

EDX mappings of the different CoW*X*00 and CoNiW*X*00 tungsten oxides show a homogenous distribution of Co/Ni, W and O in all samples (Fig. S23–S28 and Tables S5–S10†) and the elemental ratios of the CoW*X*00 series are in line with CoWO_4_.

### 3.2 Photo- and electrocatalytic water oxidation activity of Co(1) and CoNi(2)

#### 3.2.1 Photocatalytic activity of Co(1) and CoNi(2)

The photocatalytic water oxidation activities of Co(1) and CoNi(2) were investigated in a borate buffer solution (0.1 M, pH 8, 8 mL) with [Ru(bpy)_3_]^2+^ (1 mM) as photosensitizer (PS) and Na_2_S_2_O_8_ (5 mM) as sacrificial electron acceptor under irradiation at 470 nm. O_2_ evolution was monitored by GC-MS to determine the overall TON and with a Clark electrode to determine the initial TOF.

The general mechanism of photocatalytic water oxidation using the [Ru(bpy)_3_]^2+^/S_2_O_8_^2−^ assay has been studied in numerous works, which are summarized in recent topical reviews,^94^ including POM water oxidation catalysts.^15^ In short, a wide range of studies confirmed that the photoexcited state [Ru(bpy)_3_]^2+^* is quenched by S_2_O_8_^2−^ to generate [Ru(bpy)_3_]^3+^ along with 
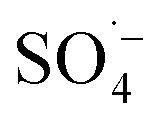
, which can bring forward another molecule of [Ru(bpy)_3_]^3+^. To finally generate O_2_, four holes are first transferred to the POM-WOC *via* four [Ru(bpy)_3_]^3+^ equivalents, and the so oxidized POM catalyst can then further oxidize two water molecules. The precise local mechanisms at the active transition metal centers of different POM-WOCs are subject to advanced theoretical studies and further investigations, and they may vary individually for each POM type.^95^

For photocatalytic performance evaluation, first Co(1) was tested in different buffer solutions and pH values to explore the optimal water oxidation conditions. Borate buffer (0.1 M, pH 8) led to the best performance ahead of borate buffer (0.1 M, pH 9) and phosphate buffer (0.1 M, pH 7). No activity was observed in acetate buffer (0.1 M, pH 4.75) (Fig. S33†).

**Fig. 4:**
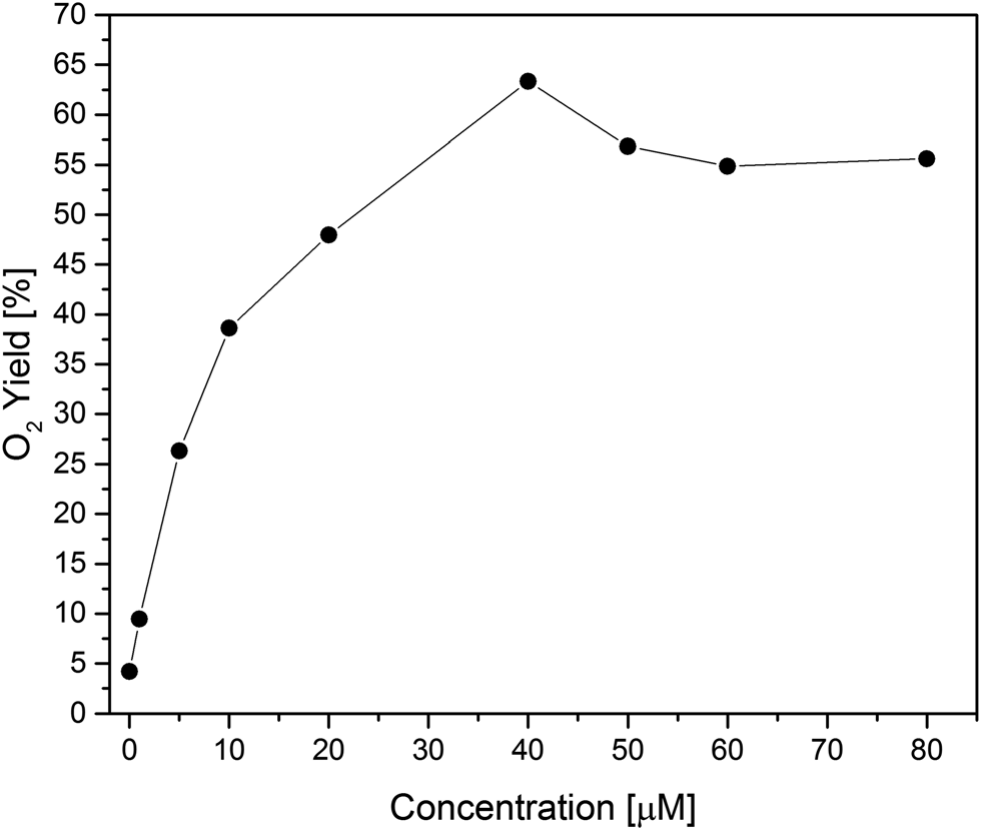
Photocatalytic oxygen yield *vs.* WOC concentration for Co(1).

Second, concentration screening of Co(1) was performed to further optimize the working conditions (Fig. 4, S34 and S35†). Although water oxidation is generally thermodynamically favorable at higher pH values, performances at pH 8 were found to be superior to pH 9,^96^ in line with other studies.^6^ The maximum O_2_ yield of 63% was achieved with 40 μM of Co(1). Compared to other reported Co- or Ni-based POMs, this O_2_ yield is competitive for the applied photocatalytic assay (Table 1). In comparison, the Mn-based analogue [Mn^III^_3_Mn^IV^O_3_(CH_3_COO)_3_(A-α-SiW_9_O_34_)]^6−^ exhibited a rather low photocatalytic performance with 3% oxygen yield.^29^

**Table 1:** TON, TOF [s^−1^], and O_2_ yield of topically related, selected POM WOCs

Catalyst	TON	TOF	O_2_ yield/%	Ref.
Co(1)	40	0.5	63	This work
CoNi(2)	16	0.2	26	This work
a[(SiW_9_O_34_)_2_Co_8_(OH)_6_(H_2_O)_2_(CO_3_)_3_]^16−^	545	3.1	44	4
b[(SiW_9_O_34_)_2_Co_8_(OH)_6_(H_2_O)_2_(CO_3_)_3_]^16−^	1436	10	29	4
c[Co^II^_5_Co^III^_2_(mdea)_4_(N_3_)_2_(CH_3_CN)_6_(OH)_2_(H_2_O)_2_]^4−^	88	1.75	24	98
d[Co_4_(H_2_O)_2_(PW_9_O_34_)_2_]^10−^	75	5	64	99
e[Co_6_(H_2_O)_30_{Co_9_Cl_2_(OH)_3_(H_2_O)_9_(SiW_8_O_31_)_3_}]^5−^	100	0.042	—	100
f[{Co_2_Sb_2_(H_2_O)_10_(B-β-(SbW_9_O_33_)}_2_]^4−^	193	5.3	31	18
[{β-SiNi_2_W_10_O_36_(OH)_2_(H_2_O)}_4_]^24^	335	1.7	27	77

a 2 μM cat., borate buffer (80 mM, pH 9).

b 0.5 μM cat., borate buffer (80 mM, pH 9).

c 25 μM cat., borate buffer (0.2 M, pH 8).

d [Ru(bpy)_3_]^3+^ used as oxidant.

e 1.27 μM cat., borate buffer (80 mM, pH 8).

f 4 μM cat., borate buffer (80 mM, pH 8.5).

In Table 1, TON, TOF and O_2_ yields are compared to several previously reported POM-WOCs. TONs were increasing with reduced catalyst concentrations, reaching a value of 235 at a catalyst concentration of 1 μM (Table S13†). In the absence of Co(1), a background O_2_ evolution of 0.83 μmol was detected, which corresponds to 4% O_2_ yield. Additionally, a reference WOC test with the same concentration of cobalt centers was performed for cobalt acetate (40 μM based on Co), and the obtained 54% O_2_ yield was lower compared to Co(1).

Recycling experiments showed further activity of the catalytic system after adding additional Na_2_S_2_O_8_ and adjusting the pH back to 8. The 2^nd^ and 3^rd^ cycle showed O_2_ yields of 25% and 11%, respectively (Table S14 and Fig. S36†). Throughout the recycling, a continuous color change was observed from bright orange to dark green, suggesting a slow decomposition of the photosensitizer [Ru(bpy)_3_]^2+^. This shows that low photosensitizer stability associated with the formation of sulfate radicals from Na_2_S_2_O_8_ are the main reasons for the decline of O_2_ evolution.

CoNi(2) showed reduced photocatalytic oxygen evolution performance compared to pure Co(1). O_2_ yields decreased from 63% to 26% at a catalyst concentration of 40 μM (Table 1). The calculated O_2_ yield per Co center (for 40 μM catalyst) is 16% for Co(1) and 15% for CoNi(2), respectively. This underscores further that the Ni centers are most likely inactive. Our previous work on the molecular cubane water oxidation catalyst [Co_4_(dpy-C{OH}O)_4_(OAc)_2_(H_2_O)_2_](ClO_4_)_2_ showed a comparable trend towards lower O_2_ yields upon introducing Ni into the cobalt sites. This may imply that an intramolecular O–O coupling pathway between two Co–OH_*n*_ ligands prevails for O_2_ evolution from such oxocluster WOCs, as reported for Co_3_O_4_.^97^CoNi(2) furthermore displays a larger band gap (2.66 eV) than Co(1) (2.41 eV, see Fig. S7 and S8†).

**Fig. 5:**
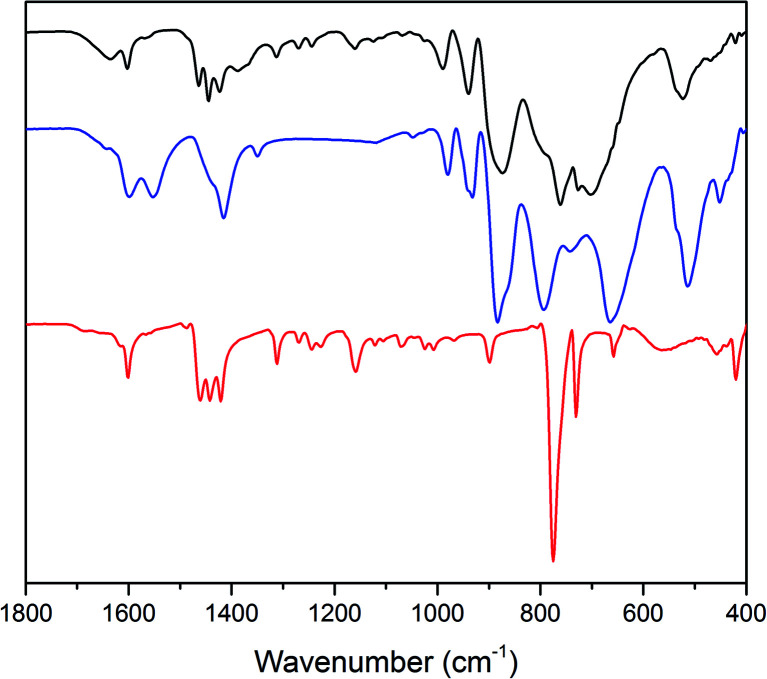
FT-IR spectra of the Co(1)–POM–PS complex (black), pristine Co(1) (blue) and [Ru(bpy)_3_]Cl_2_ (red).

The expected formation of a solid POM–PS complex was observed after the catalytic O_2_ evolution tests.^101^ FT-IR analysis shows the presence of both photosensitizer and Co(1) in the precipitate (Fig. 5). For the POM–PS complex, characteristic bands are observed at 989, 939 (*ν*_as_W–O_d_) and 874 cm^−1^ (*ν*_as_W–O_b_) compared to 980, 932 and 884 cm^−1^ for the pristine Co(1). Additional, three bands at 1463, 1444 and 1423 cm^−1^ can be attributed to [Ru(bpy)_3_]^2+^, in line with previous reports on POM–PS complex formation.^102^

This precipitation is a general phenomenon of POM-WOCs in such photocatalytic assays due to the electrostatic interactions between negatively charged POMs and positively charged photosensitizer molecules. Only at low catalyst concentrations <2 μM no measureable precipitation was detected, although such amounts most likely fall below the detection limit of frequently used DLS devices. Note that those devices were designed to quantify size distribution of large amounts of nanoparticles rather than for evidencing their absence.^103^

Further EDX analyses of the POM–PS complex show a homogeneous distribution of Ru, Co and W and calculated ratios of 2.5 : 1 [Ru(bpy)_3_]^2+^ : Co(1) (Table S4,† based on the N : W ratio, Fig. S22†). Notably, lyophilisation of the remaining solution showed no presence of cobalt, which further supports that Co(1) does not undergo leaching of Co^2+^ ions into the solution (Fig. S21†). A subsequent WOC test with the filtered solution and additional Na_2_S_2_O_8_ showed no further activity (Fig. S37†).

Photocatalytic tests with the recovered POM–PS complex, revealed its continuous activity with 34% O_2_ yield, which is superior to the direct recycling run of the pristine POM in solution. This shows that the POM–PS complex is still active as a catalyst and that the photocatalytic assay ([Ru(bpy)_3_]^2+^/Na_2_S_2_O_8_) causes the fast decline of O_2_ formation for pristine Co(1).

#### 3.2.2 Electrocatalytic activity of Co(1) and CoNi(2)

**Fig. 6:**
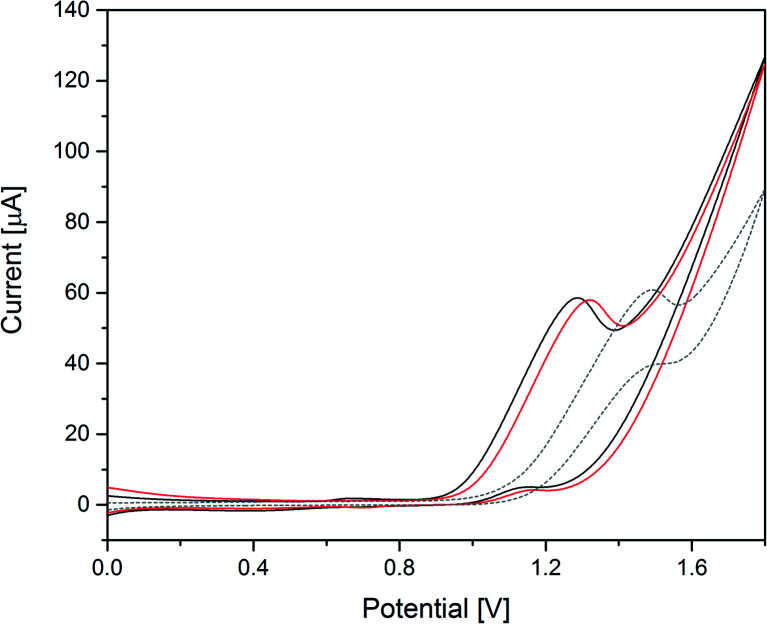
Cyclic voltammograms of 50 μM Co(1) (black) and 50 μM CoNi(2) (red) in 0.1 M borate buffer pH 8 and blank measurements (grey, dashed; V *vs.* Ag/AgCl, scan rate: 20 mV s^−1^, 3^rd^ scan is shown).

Fig. 6 displays the results of electrocatalytic activity tests, where the onset potentials for Co(1) and CoNi(2) were determined as 0.96 V and 1.00 V *vs.* Ag/AgCl, respectively. The higher onset potential of CoNi(2) corresponds to the lower photocatalytic water oxidation performance. According to previous reports, Co-based POM electrocatalysts can undergo Co^2+^ leaching, especially in basic conditions.^104^ The leached Co^2+^ ions can then form an active heterogeneous CoO_*x*_ film on the working electrode and contribute to the WOC activity.^30^ During several CV scans, Co(1) and CoNi(1) showed minor shifts of the onset potential and anodic peaks to lower potentials (0.08 V after 3 cycles, Fig. S38 and S39†), which might be attributed to such CoO_*x*_ formation on the working electrode. It is of note that the Pt counter electrode was tested in the applied potential range and showed no activity (see blank measurements below). Previous reports showed dissolution and re-deposition of Pt on the reduction half-cell in acidic reaction media.^105^ To the best of our knowledge, no such influence of Pt electrodes has been reported for the OER before.^106^

### 3.3. Co(1) and CoNi(2) as precursors for heterogeneous WOCs

Next, the heterogeneous tungstate WOCs emerging from Co(1) and CoNi(2) as precursors were investigated for their electrocatalytic performance.

**Fig. 7:**
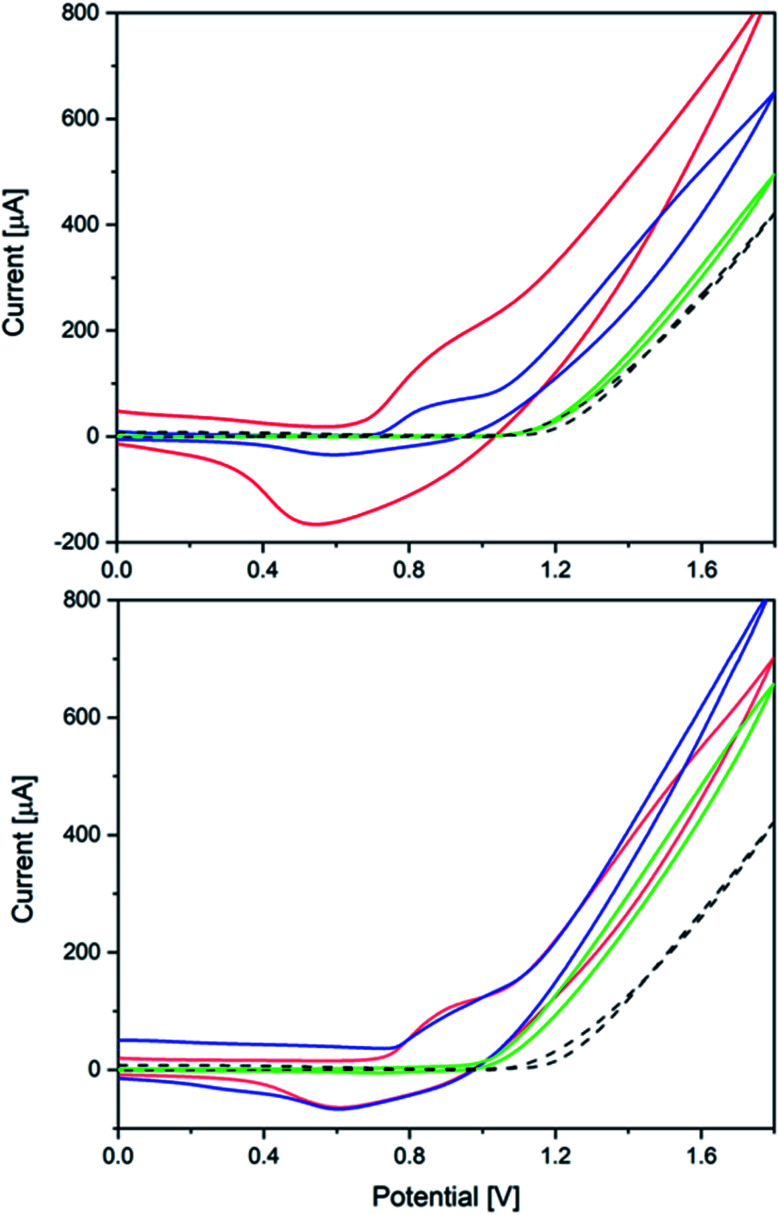
Top: Cyclic voltammograms of CoW300 (red), CoW400 (blue) and CoW500 (green); bottom: CVs of CoNi300 (red), CoNi400 (blue) and CoNi500 (green); all measurements on FTO in 0.1 M borate buffer pH 8 *vs.* blank measurements (black, dashed; V *vs.* Ag/AgCl, scan rate: 20 mV s^−1^, 3^rd^ scan is shown).

The significant influence of the annealing temperature is quite evident from the cyclic voltammetry results (Fig. 7). Amorphous CoW300 has an onset potential of 0.79 V *vs.* Ag/AgCl in borate buffer (0.1 M, pH 8) solution and maintains its catalytic performance over the measured eight cycles.

A broad cathodic peak at 0.55 V can be seen in the backward scan, which moves slowly to higher potentials in the subsequent scans (0.07 V during eight cycles, *cf.* Fig. S47†). The amorphous sample shows constant catalytic performance over several cycles with stable anodic and cathodic peaks. Previously reported amorphous CoWO_4_ showed a substantial change in the anodic and cathodic peak after the first cycle.^66^

The onset potential of the crystalline samples CoW400 and CoW500 gradually increases to 0.94 V and 1.18 V *vs.* Ag/AgCl, respectively (Fig. S45†). This is in line with previous observations for amorphous and crystalline CoWO_4_ and their onset potentials.^68,69^ Interestingly, the onset potential of CoW500 is almost the same as of the blank FTO electrode.

The onset potentials for the Ni-doped analogues are shifted to higher potentials (Fig. 7). Other than the pure Co samples, the onset potential values for CoNi300 and CoNi400 are closer, namely 0.91 V and 0.98 V, respectively (Fig. S46†). On the other hand, CoNi500 has a better onset potential (1.08 V) compared to its binary analogue CoW500 (1.18 V).

As mentioned above, the blue shift of the {WO_4_} Raman peak of CoW300 suggests a reduced distance between Co^2+^ ions, which can further influence the mechanism of the electrocatalytic water oxidation.^66,92^ This was previously reported and confirmed with EXAFS analyses for amorphous and crystalline CoWO_4_,^33^ where the outstanding performance of the amorphous CoWO_4_ was attributed to the shorter Co–Co distances.

Although the water oxidation reaction is a complex process, it was reported in previous studies that its mechanism strongly depends on the distance between the active sites of heterogeneous electrocatalysts.^33^ Along these lines, it is assumed that closer intermetallic distances in the range of the O–O bond distance of dioxygen are favoring the “dual-site” Langmuir–Hinshelwood (LH) mechanism. In this mechanism, oxygen species are first adsorbed on adjacent sites, followed by their formation of molecular oxygen. In the case of longer distances between the active metal sites, however, the “single-site” Eley–Rideal (ER) mechanism may take place instead.

The dual-site LH mechanism requires a lower overpotential than the single-site ER mechanism, because the latter includes the formation of a peroxo intermediate at the single active metal center. This is considered a thermodynamically less favorable step for the overall water oxidation.^66,107^ In the present case, the observed decrease of the Co–Co distances in CoW300 may therefore facilitate the bridging of two terminal oxo groups to generate dioxygen *via* the LH mechanism.^66,108^ This agrees with the observed lowest onset potential for CoW300 among the tungstate catalyst series.

**Fig. 8:**
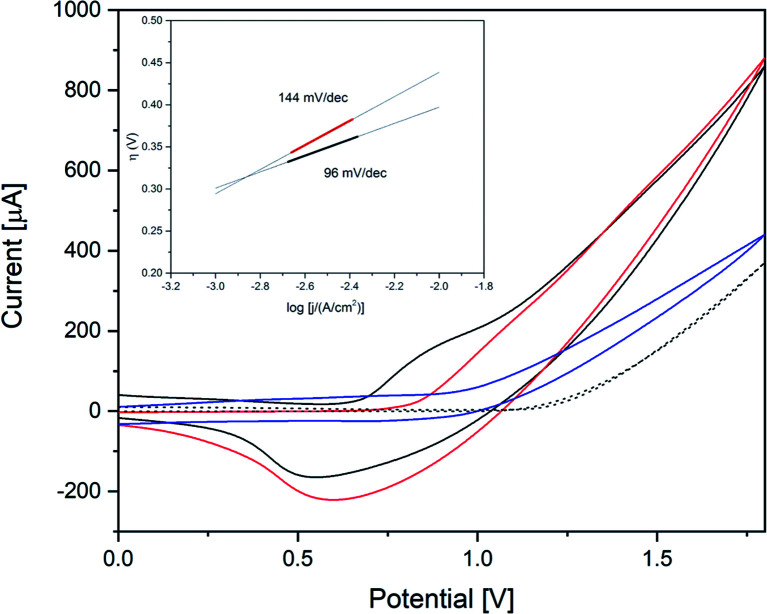
Cyclic voltammograms of CoW300 (black); CoWO_4_ (red); RuO_2_ (blue) and reference FTO (dotted) in 0.1 M borate buffer pH 8 (scan rate: 20 mV s^−1^); inset: Tafel plot of CoW300 (black) and CoWO_4_ (red).

CoW300 as best performing member of the tungstate series was further compared to a reference sample obtained from a conventional solution co-precipitation method (annealed at 300 °C) for CoWO_4_ and to RuO_2_ as a well-established benchmark WOC (Fig. 8).^66^CoW300 showed a lower onset potential (0.79 V) than the as-synthesized reference sample (0.84 V).

Furthermore, chronoamperometry measurements of CoW300 and the conventionally synthesized reference material were performed. The superior performance of CoW300 is clearly evident from the lower Tafel slope (inset Fig. 8) of 96 mV dec^−1^, compared to 144 mV dec^−1^ for the reference material obtained from Na_2_WO_4_ and Co(NO_3_)_2_.^66^

## 4. Conclusions

A bio-inspired polyoxometalate with an open Co_4_-core architecture, K_5_Na_3_[(A-α-SiW_9_O_34_)Co_4_(OH)_3_(CH_3_COO)_3_] Co(1), was synthesized as a model system to investigate crucial questions of water oxidation catalyst (WOC) design, namely (1) the controversially discussed effect of Co/Ni-synergisms in molecules *vs.* solids, (2) the influence of preparative history and precursor choice on WOC activity and (3) the role of amorphous features in solid WOC performance.

First, the open Co_4_-POM Co(1) displayed competitive photocatalytic activity with an oxygen yield of 63% for the optimal catalyst concentration of 40 μM. Furthermore, its new mixed Co/Ni isostructural analogue CoNi(2) was synthesized, analyzed and tested for water oxidation activity. Co/Ni substitution did not exert a productive influence on the water oxidation activity of the mixed-metal POMs, in contrast to widely reported Co/Ni synergisms in solid WOCs.

Next, to investigate the effect of mixed metal molecular precursors on cobalt tungstate-related WOCs as attractive target materials, Co(1) and CoNi(2) were subjected to thermal treatment. Both compounds afforded CoWO_4_- as well as (Co, Ni)WO_4_-related phases with increasing degrees of crystallinity upon higher annealing temperatures.

Concerning the influence of crystallinity on the performance, cyclic voltammetry measurements clearly showed that amorphous CoW300 obtained from annealing Co(1) at 300 °C showed the lowest onset potential among both series of tungsten oxides obtained from POM precursors. Most importantly, CoW300 displayed a lower onset potential than a representative reference sample of CoWO_4_ that was synthesized *via* a conventional co-precipitation/annealing method.

In line with the catalytic trends for the Co(1) and CoNi(2) precursor POMs, introduction of nickel centers did not exert a productive effect on the heterogeneous tungstate catalysts either. This is in stark contrast to the growing number of literature reports on Co/Ni synergisms in a wide range of oxide and non-oxide heterogeneous electrocatalysts. Further systematic studies are now required to understand the dependence of such metal–metal interactions on the catalyst matrix and its preparative history, as well as on the applied performance test conditions.

Our results demonstrate that readily accessible POMs are promising precursors with pre-organized metal centers for the convenient synthesis of amorphous heterogeneous water oxidation catalysts, which outperform products of conventional high temperature approaches starting from simple binary educts. Interestingly, neither the molecular precursors nor their heterogeneous WOC products were responsive to widely employed synergistic Co/Ni doping strategies. This highlights the complexity and matrix dependence of such mixed metal optimization strategies, which are in the focus of forefront catalytic endeavours.

To fully transfer the tunable potential of polynuclear molecular precursors into high performance amorphous catalysts with optimal near-range order properties, in-depth monitoring and theoretical studies of mixed metal interactions in different settings are now required.

## Conflicts of interest

There are no conflicts of interest to declare.

## Supplementary Material

RA-011-D0RA10792A-s001
