# Supplementary material for: Co/Ni-polyoxotungstate photocatalysts as precursor materials for electrocatalytic water oxidation
Source: RSC Adv. 2021 Mar 18;11(19):11425–36. doi: 10.1039/d0ra10792a (PMC8695939; doi:10.1039/d0ra10792a)
Supplement: RA-011-D0RA10792A-s001 [file RA-011-D0RA10792A-s001.pdf]

This journal is © The Royal Society of Chemistry 2021

Co- and Co/Ni-Polyoxotungstate Photocatalysts as Precursors for Electrocatalytic Water Oxidation Routes

Robin Güttinger, Giann Wiprächtiger, Olivier Blacque, and Greta R. Patzke*

Supporting Information

Table of Contents

1. Characterization of Co(1), CoNi(2), CoWX00 and CoNiX00

Figure S1. FT-IR spectrum of **Co(1)**. 5

Figure S2. FT-IR spectrum of **CoNi(2)**. 5

Figure S3. FT-IR spectrum of the **Co(1)**-POM-PS complex. 6

Figure S4. FT-IR spectra of **CoW300** (red), **CoW400** (green), and **CoW500** (blue). 6

Figure S5. FT-IR spectra of **CoNi300** (red), **CoNi400** (green), and **CoNi500** (blue). 7

Figure S6. UV/vis absorption spectrum of **Co(1)** (20 μM) in borate buffer over 24 h and 5 h 7

Figure S7. Band gap determination of **Co(1)** 8

Figure S8. Band gap determination of **CoNi(2)** 8

Figure S9. Raman spectrum of **Co(1)**. 9

Figure S10. Raman spectrum of **CoNi(2)**. 9

Figure S11. Raman spectrum of the **Co(1)**-POM-PS complex. 10

Figure S12. Raman spectrum of conventionally synthesized $CoWO_4$ at 300 °C 10

Figure S13. Rietveld refinement of the PXRD pattern of **Co(1)** 11

Figure S14. PXRD pattern of **CoNi(2)** vs. calculated patterns of **Co(1)** and {Ni_4} 11

Figure S15. Rietveld refinement of the PXRD pattern of **CoNi(2)** 12

Figure S16. Peak shift in the PXRD patterns of **CoNi500** vs. **CoW500** 12

Figure S17. PXRD pattern of conventionally synthesized **$CoWO_4$** at 300 °C 14

Table S1. Rietveld refinement results of **Co(1)**. 13

Table S2. Rietveld refinement results of **CoNi(2)**. 13

Figure S18. TG characterization of **Co(1)**. 15

Figure S19. TG characterization of **CoNi(2)**. 15

Figure S20. EDX spectrum of **CoNi(2)**. 16

Table S3. EDX analysis of **CoNi(2)** 16

Figure S21. EDX of lyophilized solution after Clark electrode tests of **Co(1)**. 17

Figure S22. EDX-mapping of **Co(1)**-POM-PS complex....18
Table S4. EDX analysis of **Co(1)**-POM-PS complex....18
Figure S23. EDX mapping of **CoW300**....19
Table S5. EDX analysis of **CoW300**....19
Figure S24. EDX mapping of **CoW400**....20
Table S6. EDX analysis of **CoW400**....20
Figure S25. EDX mapping of **CoW500**....21
Table S7. EDX analysis of **CoW500**....21
Figure S26. EDX mapping of **CoNi300**....22
Table S8. EDX analysis of **CoNi300**....22
Figure S27. EDX mapping of **CoNi400**....23
Table S9. EDX analysis of **CoNi400**....23
Figure S28. EDX mapping of **CoNi500**....24
Table S10. EDX analysis of **CoNi500**....24
Table S11. ICP-MS analyses of pristine and calcination products of POM precursors....25
Table S12. XPS analysis of **CoNi(2)**....25
Figure S29. HR-ESI-MS of **Co(1)**-POM....26
Figure S30. HR-ESI-MS of **Co(1)**-POM....26
Figure S31. HR-ESI-MS of **CoNi(2)**....26
Figure S32. HR-ESI-MS of **CoNi(2)**....27

2. *Photocatalytic performance of Co(1) and CoNi(2)*

Figure S33. Clark-electrode kinetics of visible-light-driven O_2 evolution with **Co(1)** in different buffer conditions....28
Figure S34. Clark-electrode kinetics of visible-light-driven O_2 evolution with different concentrations of **Co(1)**....28
Figure S35. Clark-electrode kinetics of visible-light-driven O_2 evolution for different concentrations of **Co(1)**....29
Table S13. Catalytic activity of **Co(1)** over the concentration range 0 – 100 μM....29
Figure S36. Clark-electrode kinetics of visible-light-driven O_2 evolution recycling test with **Co(1)**....30
Figure S37. Clark-electrode kinetics of O_2 evolution filter test with **Co(1)**....31
Table S14. Catalytic activity of **Co(1)** in three recycling tests (40 μM WOC)....30

3. Electrochemical characterization of Co(1), CoNi(2), CoWX00 and CoNiX00

Figure S38. Cyclic voltammogram of **Co(1)** in borate buffer (0.1 M, pH 8). 32

Figure S39. Cyclic voltammogram of **CoNi(2)** in borate buffer (0.1 M, pH 8) 32

Figure S40. Cyclic voltammogram of **Co(1)**, $[Ru(bpy)_3]Cl_2$ and $Na_2S_2O_8$ in borate buffer ... 33

Figure S41. Cyclic voltammogram of $[Ru(bpy)_3]Cl_2$ and $Na_2S_2O_8$ in borate buffer pH 8 33

Figure S42. Cyclic voltammogram of $[Ru(bpy)_3]Cl_2$ in borate buffer pH 8. 34

Figure S43. Cyclic voltammogram of borate buffer pH 8 34

Figure S44. CV of **Co(1)**-POM-PS complex in borate buffer pH 8. 35

Figure S45. Cyclic voltammogram of **CoW300**, **CoW400**, **CoW500** 35

Figure S46. Cyclic voltammogram of **CoNi300**, **CoNi400**, **CoNi500** 36

Figure S47. Cyclic voltammogram of **CoW300** 36

Figure S48. Cyclic voltammogram of **CoNi300** 37

Single-crystal X-ray diffraction data were collected at 183(1) K on a *Rigaku Oxford Diffraction Xcalibur* Ruby area-detector diffractometer using a single wavelength Enhance X-ray source with Mo K_α radiation (λ = 0.71073 Å)[1] from a micro-focus X-ray source and an *Oxford Instruments Cryojet XL* cooler. Selected suitable single crystals were mounted using polybutene oil on a flexible loop fixed on a goniometer head and immediately transferred to the diffractometer. Pre-experiment, data collection, data reduction and analytical absorption correction[2] were performed with the program suite *CrysAlisPro*.[3] Using *Olex2*,[4] the structures were solved with the SHELXT[5] small molecule structure solution program and refined with the *SHELXL2014/7* program package[6] by full-matrix least-squares minimization on F^2. *PLATON*[7] was used to check the results of the X-ray analysis. A raw data set was recorded to verify the structural identity of **Co(1)** in addition to Rietveld refinement data.

Visible-light-driven water oxidation was performed in a 10 mL headspace vial sealed with an aluminum crimp cap with a rubber septum (PTFE). Reaction mixtures were prepared under dark conditions as follows: 8 mL buffered solution (e.g. borate buffer (0.1 M, pH 8)) containing 1 mM $[Ru(bpy)_3]Cl_2$, 5 mM $Na_2S_2O_8$ and the respective concentration of catalyst concentration were added to a glass vial. The solution was deaerated by purging with helium (6.0 purity) for 30 min. After purging, an initial sample of the headspace (100 µL) was injected into a gas chromatograph as background reference for the GC calibration. Contamination of the headspace by air was constantly quantified by measuring the N_2 peak on GC chromatograms. Calibration was performed by injection of known quantities of pure oxygen diluted in the same headspace vial containing the same volume and concentration of buffered solution as used for the measurements. After background calibration of the GC, the Clark-type O_2 probe was introduced into the catalytic vial, first into the headspace until the signal remained constant, and afterwards below the liquid surface accordingly.

All experiments were conducted under controlled stirring (750 rpm). Data collection was performed with the SensorTrace software from Unisense at a frequency of 1 data point per sec for both probes (temperature and oxygen). After a constant signal for the O_2 sensor was recorded, the catalytic reaction was initiated by exposing the reaction vial to the light of a 470 nm high flux LED from Rhopoint Components LTD. LED power was determined as 26.1 mW/cm^2. **Kinetic evaluations** were performed for the initial phase of approximately linear time-dependent increase of O_2 evolution (0 – 120 sec) to exclude the influence of oxygen diffusion into the headspace at a later stage.

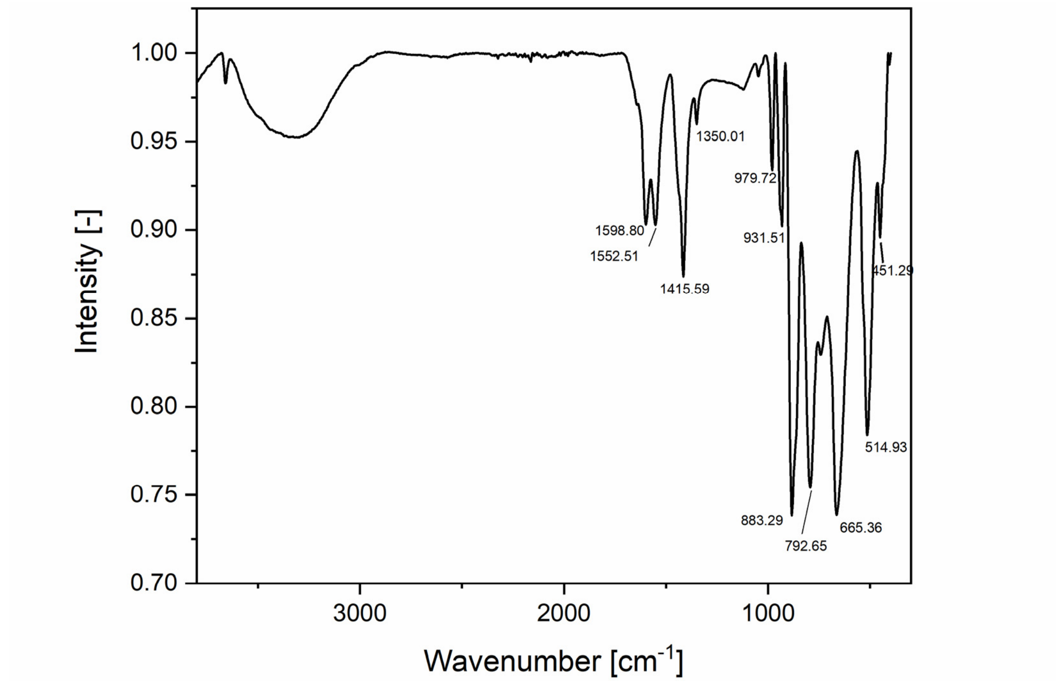

Figure S1. FT-IR spectrum of **Co(1)**.

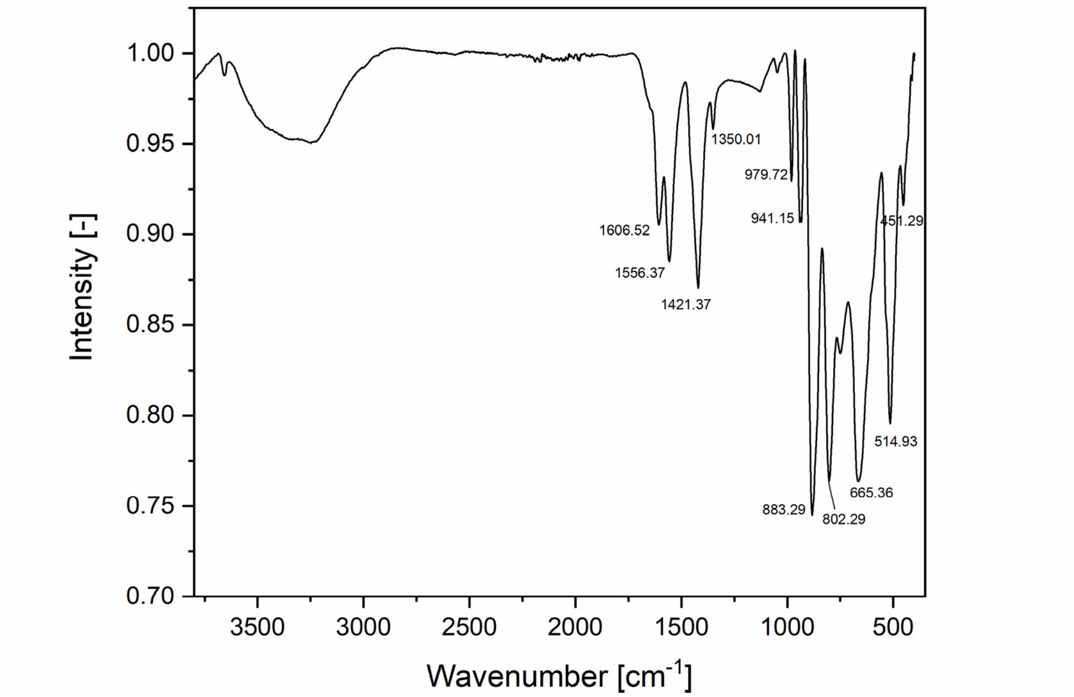

Figure S2. FT-IR spectrum of **CoNi(2)**.

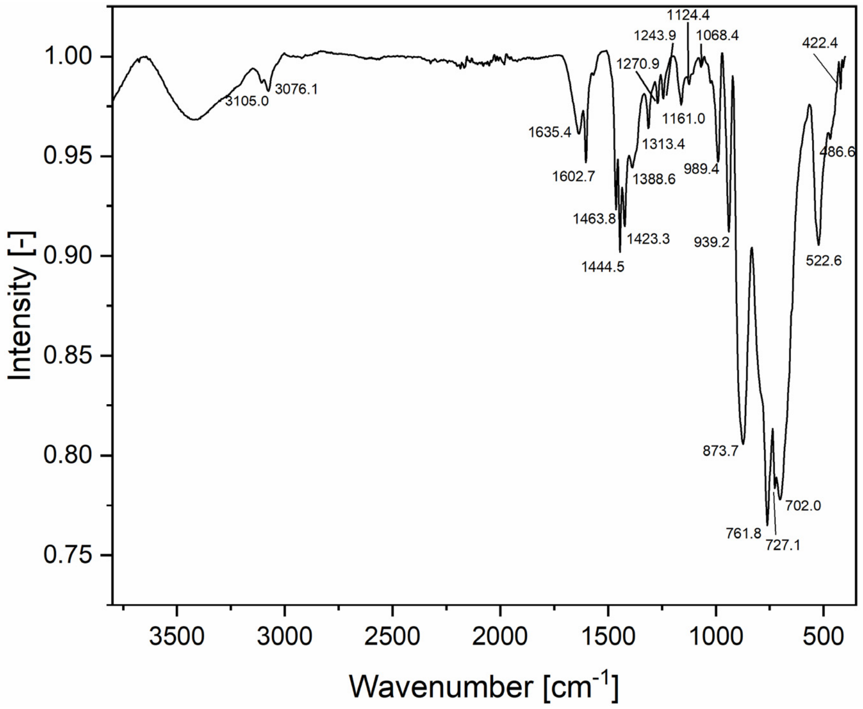

Figure S3. FT-IR spectrum of the **Co(1)**-POM-PS complex.

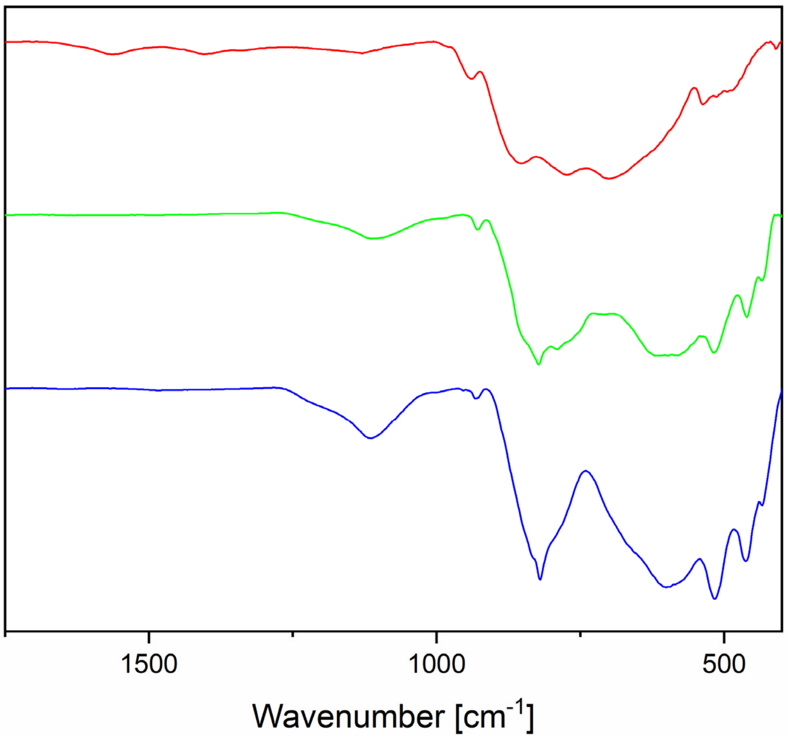

Figure S4. FT-IR spectra of **CoW300** (red), **CoW400** (green), and **CoW500** (blue).

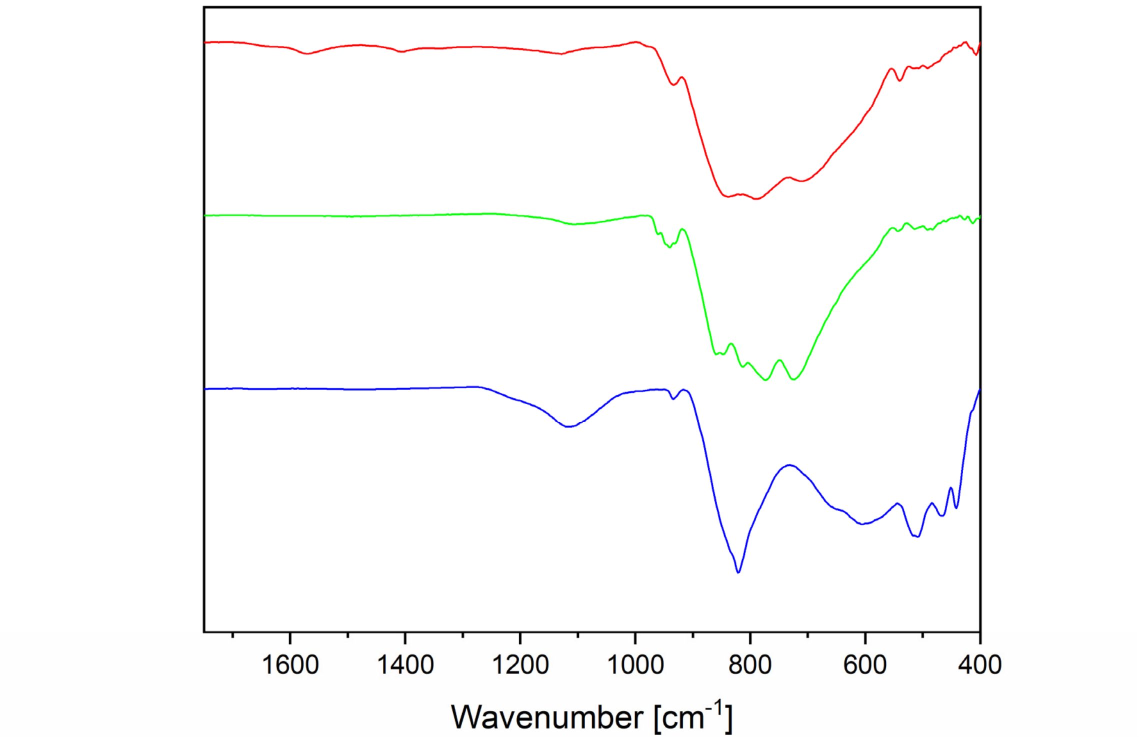

Figure S5. FT-IR spectra of **CoNi300** (red), **CoNi400** (green), and **CoNi500** (blue).

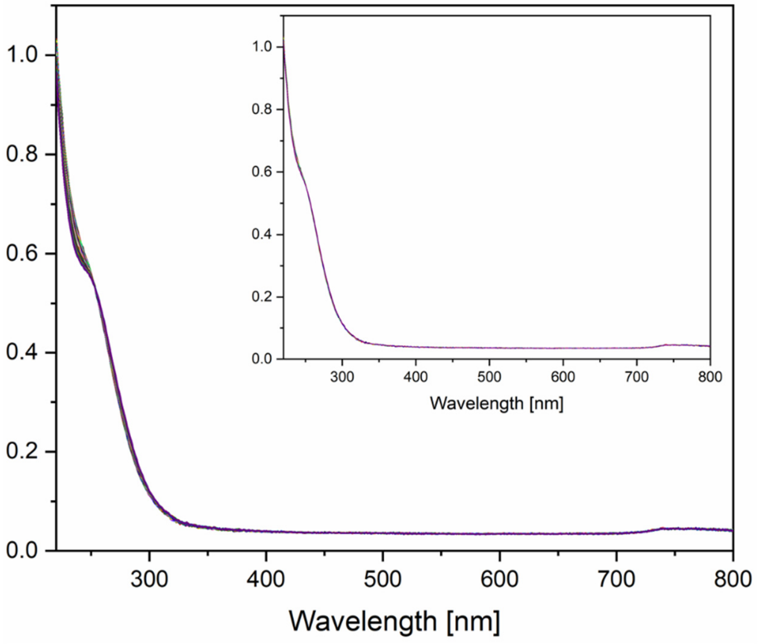

Figure S6. UV/vis absorption spectrum of **Co(1)** (20 µM) in borate buffer pH 8 (0.1 M) showing no reduction of absorbance over 24 h (inset: development of the UV/vis spectra over 5 h).

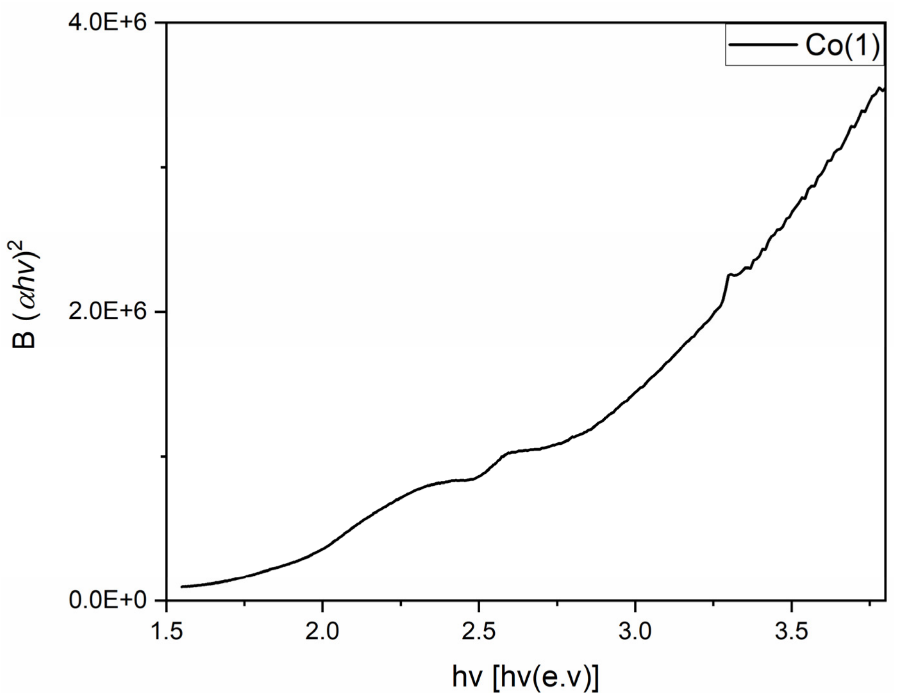

Figure S7. Band gap determination of **Co(1)** (2.41 eV).

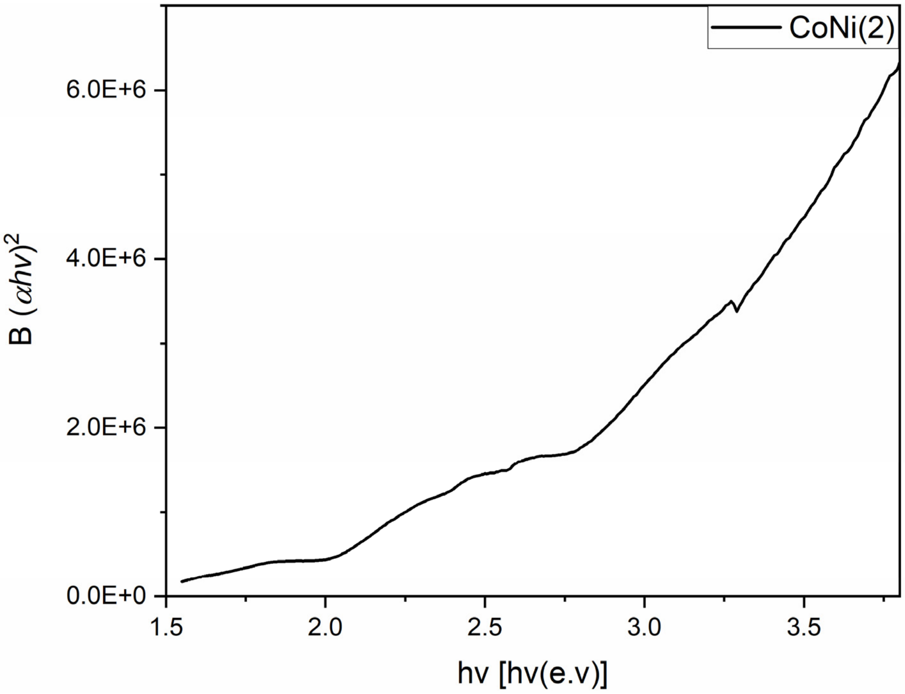

Figure S8. Band gap determination of **CoNi(2)** (2.66 eV)

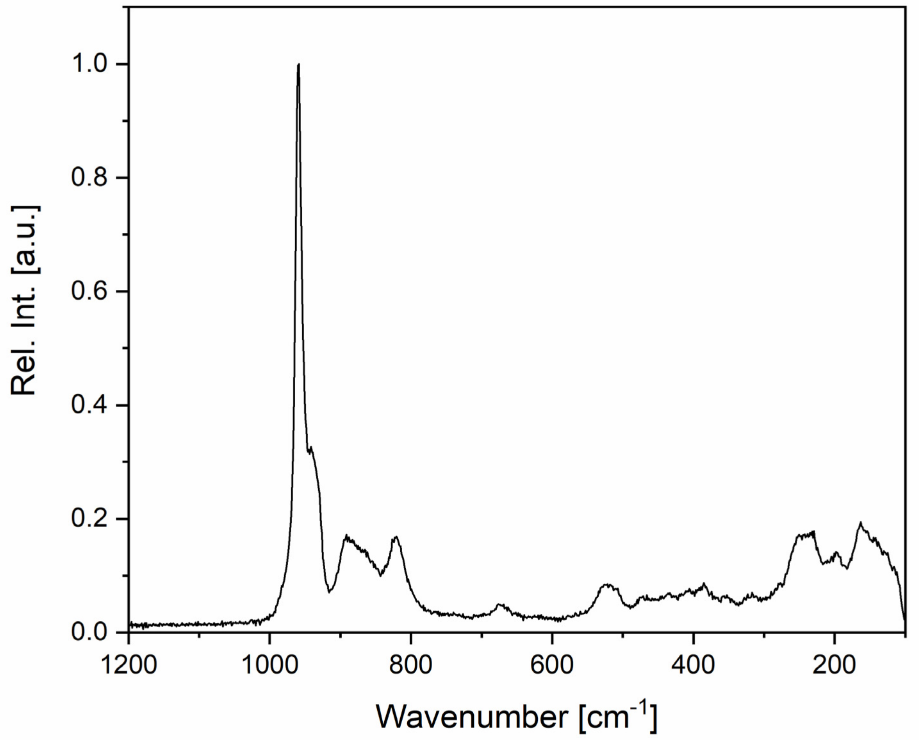

Figure S9. Raman spectrum of **Co(1)**.

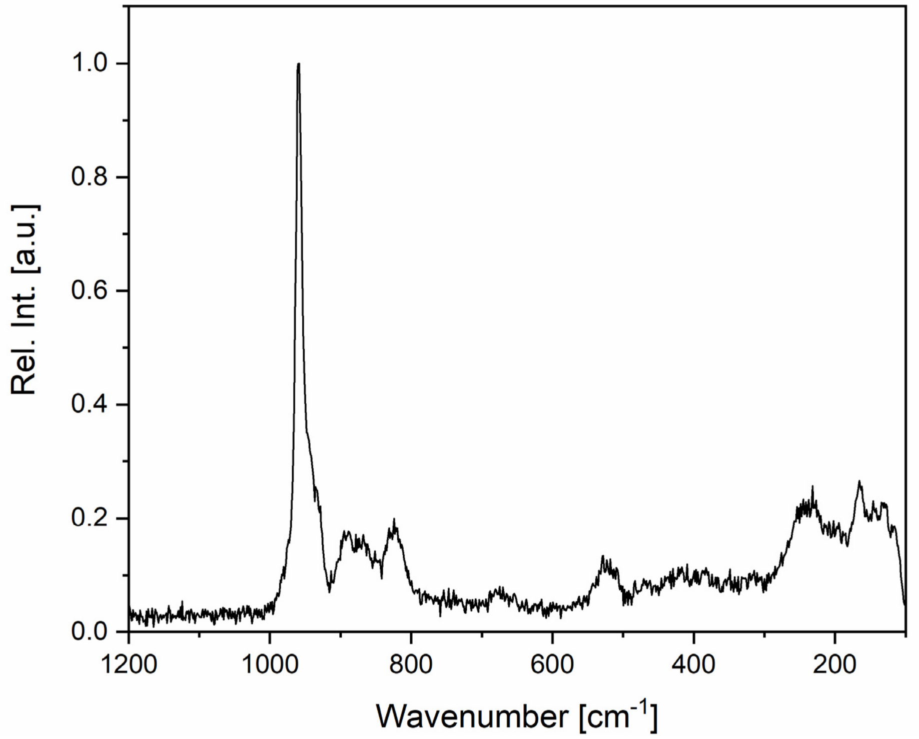

Figure S10. Raman spectrum of **CoNi(2)**.

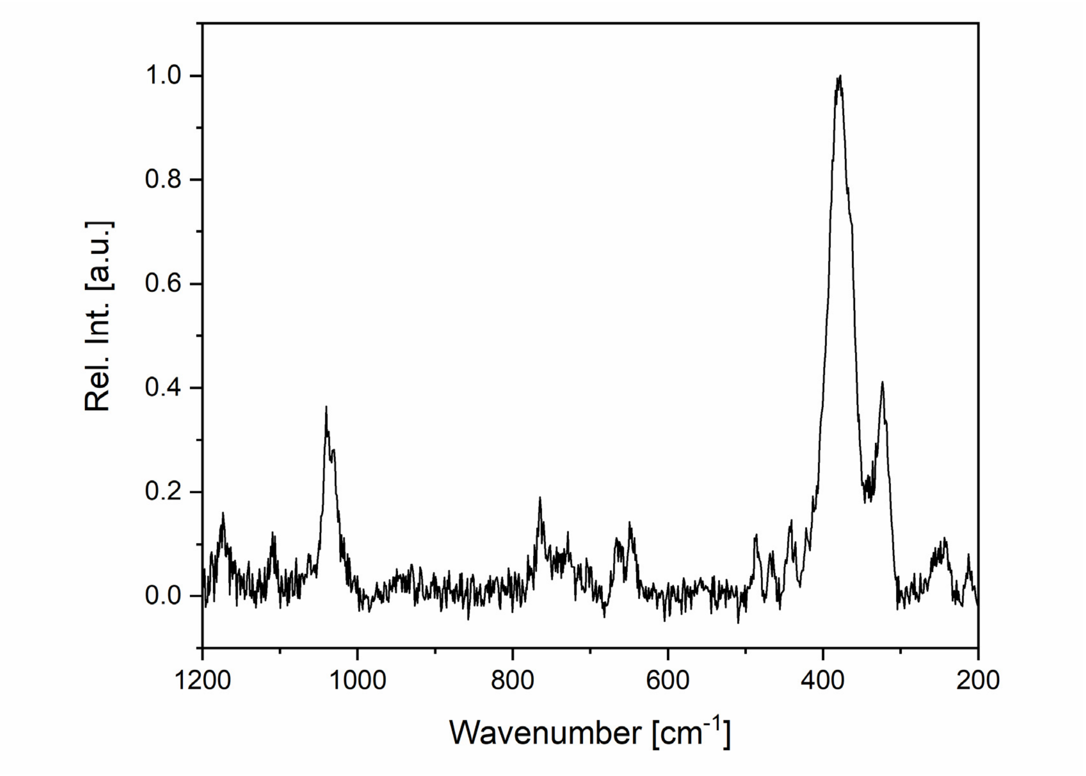

Figure S11. Raman spectrum of the **Co(1)**-POM-PS complex.

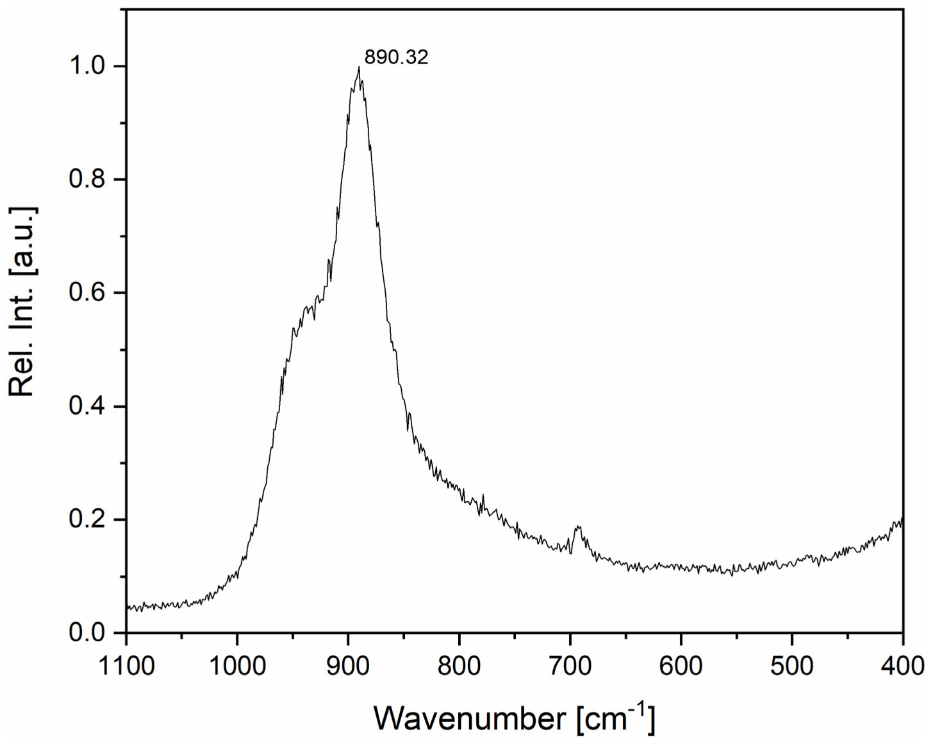

Figure S12. Raman spectrum of conventionally synthesized $CoWO_4$ annealed at 300 °C.[8]

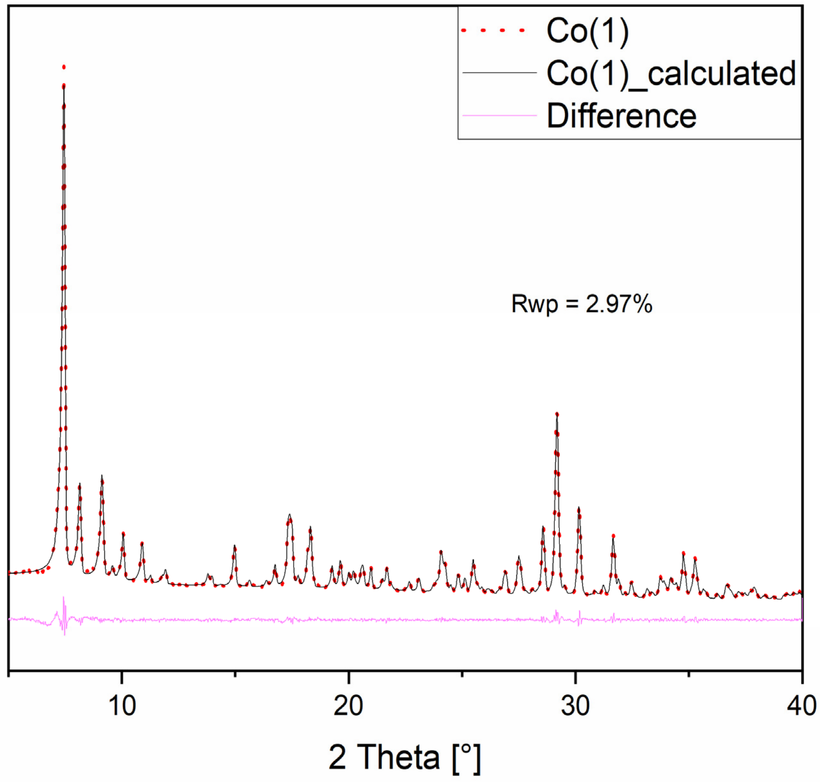

Figure S13. Rietveld refinement of the PXRD pattern of **Co(1)** vs. literature data (CCDC-619251).

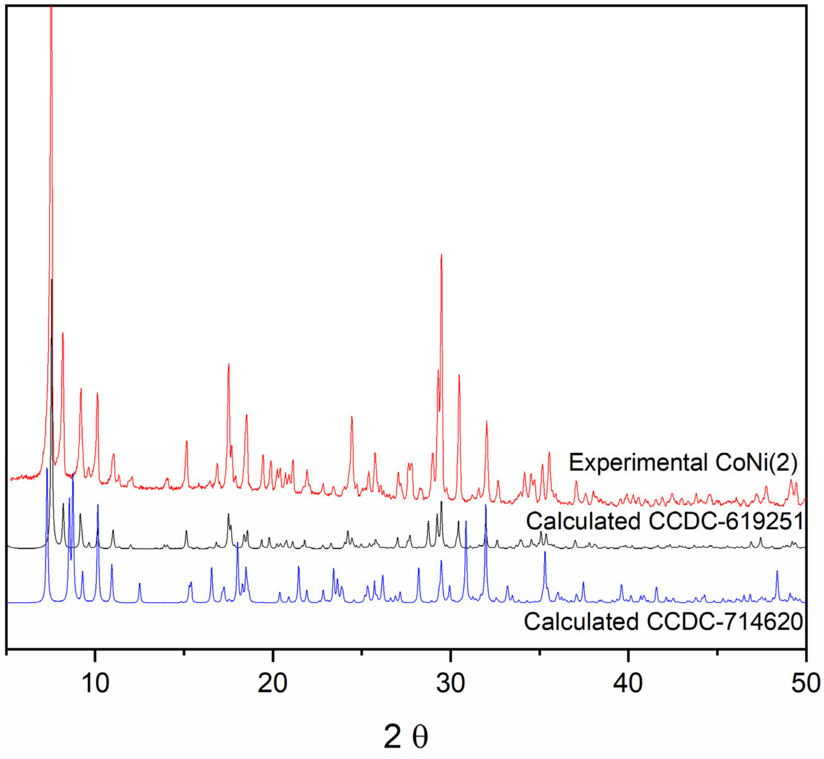

Figure S14. PXRD pattern of **CoNi(2)** vs. calculated patterns of **Co(1)** (CCDC-619251, red) and of its $\{Ni_4\}$ analogue (CCDC-714620, blue).

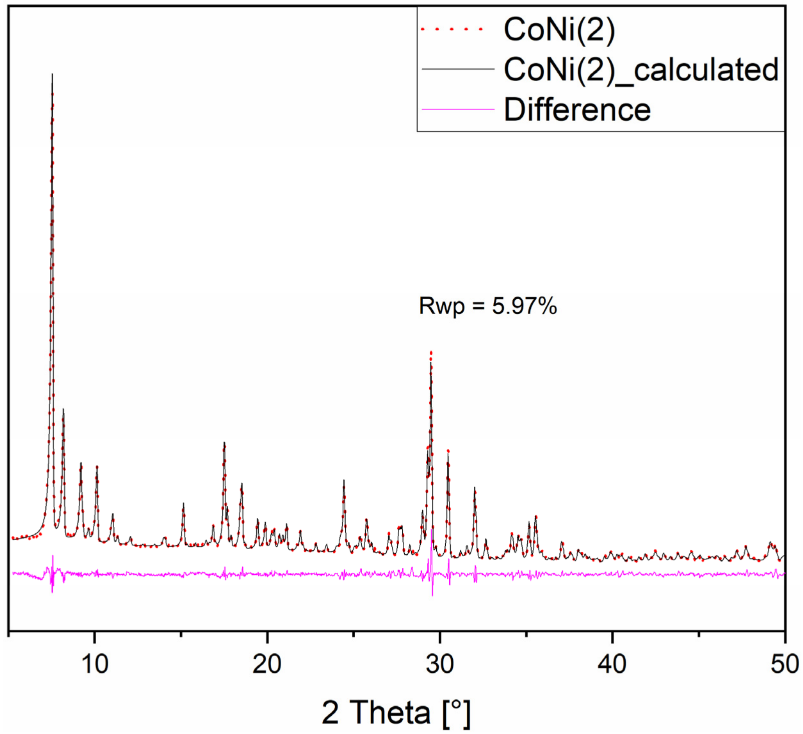

Figure S15. Rietveld refinement of the PXRD pattern of **CoNi(2)** vs. literature data (CCDC-619251).

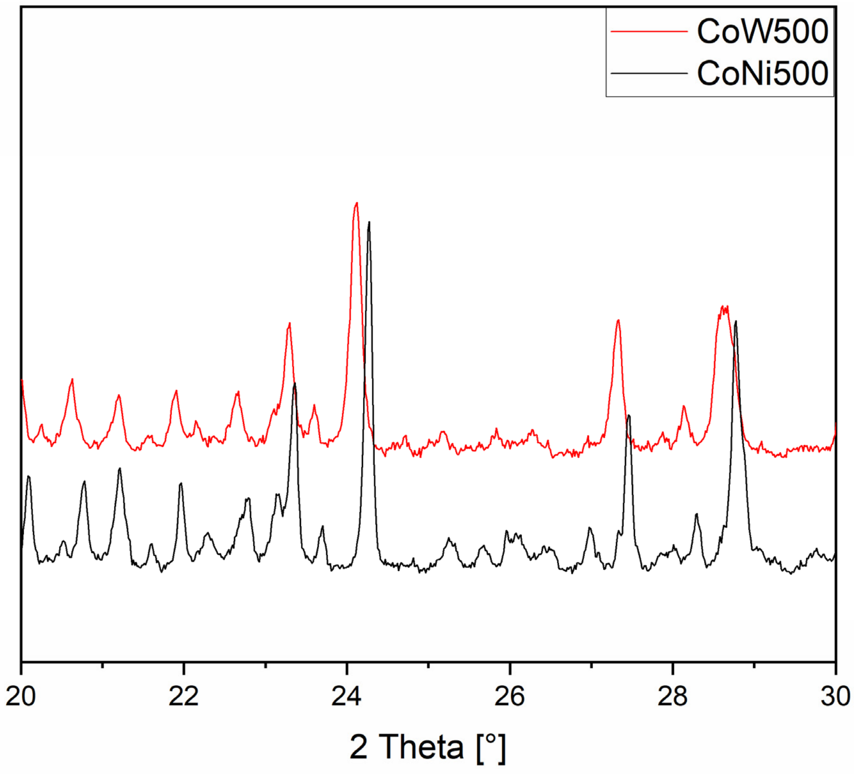

Figure S16. Peak shift in the PXRD patterns of **CoNi500** (black) vs. **CoW500** (red) (selected area of the pattern shown).

Table S1. Rietveld refinement results of **Co(1)**.

**Formula**	C6 H46 Co4 K5 Na3 O60 Si W9
**Figure of merit**	0.865
**Database entry**	CCDC-619251
a **(Å)**	10.8159(7)
b **(Å)**	14.9774(10)
c **(Å)**	19.2353(14)
**alpha (deg)**	90
**beta (deg)**	93.390(4)
**gamma (deg)**	90
**V ($Å^3$)**	3110.5(4)
**S.G.**	*P2/m*
**Z, Z’**	2, 0.5
**Calc. density (g/cm^3)**	3.391

Table S2. Rietveld refinement results of **CoNi(2)**.

**Formula**	C6 H46 Co1.5 Ni2.5 K5 Na3 O60 Si W9
**Figure of merit**	0.591
**Database entry**	CCDC-619251
a **(Å)**	10.7603(6)
b **(Å)**	14.8133(7)
c **(Å)**	18.9649(11)
**alpha (deg)**	90
**beta (deg)**	93.156(3)
**gamma (deg)**	90
**V ($Å^3$)**	3018.4(3)
**S.G.**	*P2/m*
**Z, Z’**	2, 0.5
**Calc. density (g/cm^3)**	3.478

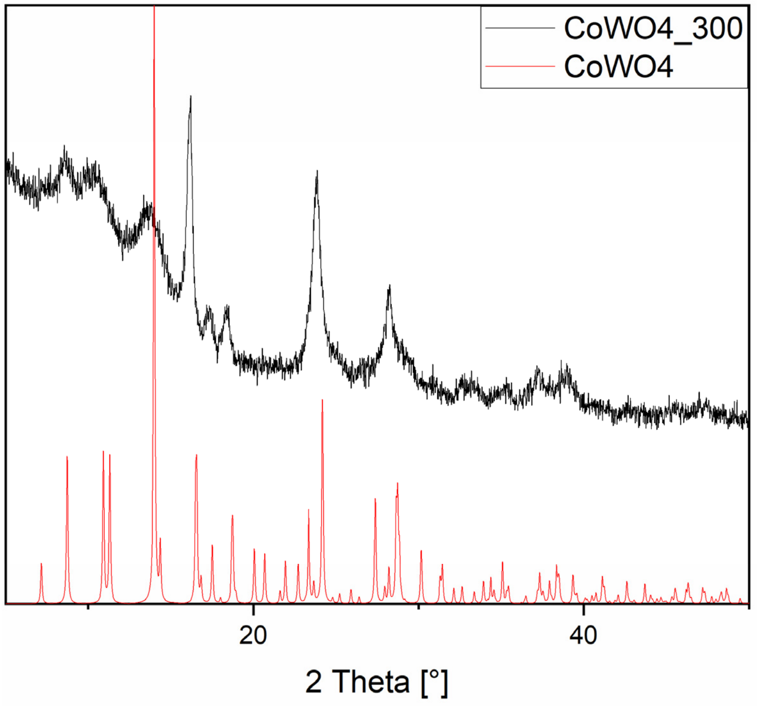

Figure S17. PXRD pattern of conventionally synthesized **$CoWO_4$** (black) obtained from annealing of $Co(NO_3)_2/Na_2WO_4$ precipitate at 300 °C vs. calculated pattern of $CoWO_4$ (CCDC-619251, red).[8]

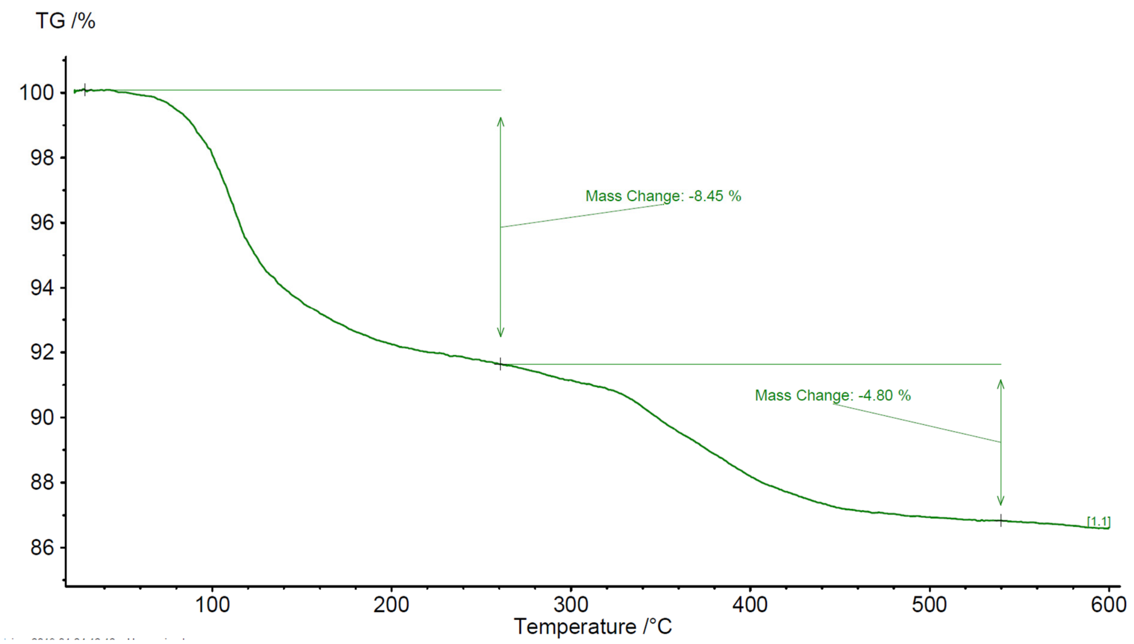

Figure S18. TG characterization of **Co(1)**.

In the temperature range between 24 and 260 °C, a mass loss step of 8.45% indicates the loss of 15 water molecules. A further mass loss step at 260 °C is related to the onset of CO_x evolution.

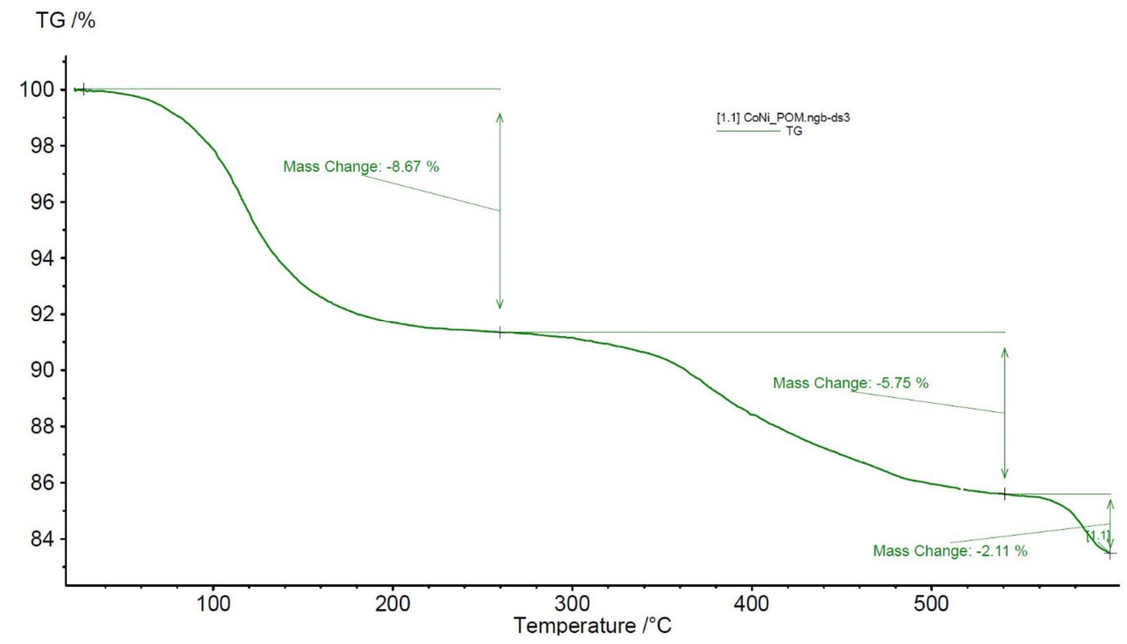

Figure S19. TG characterization of **CoNi(2)**.

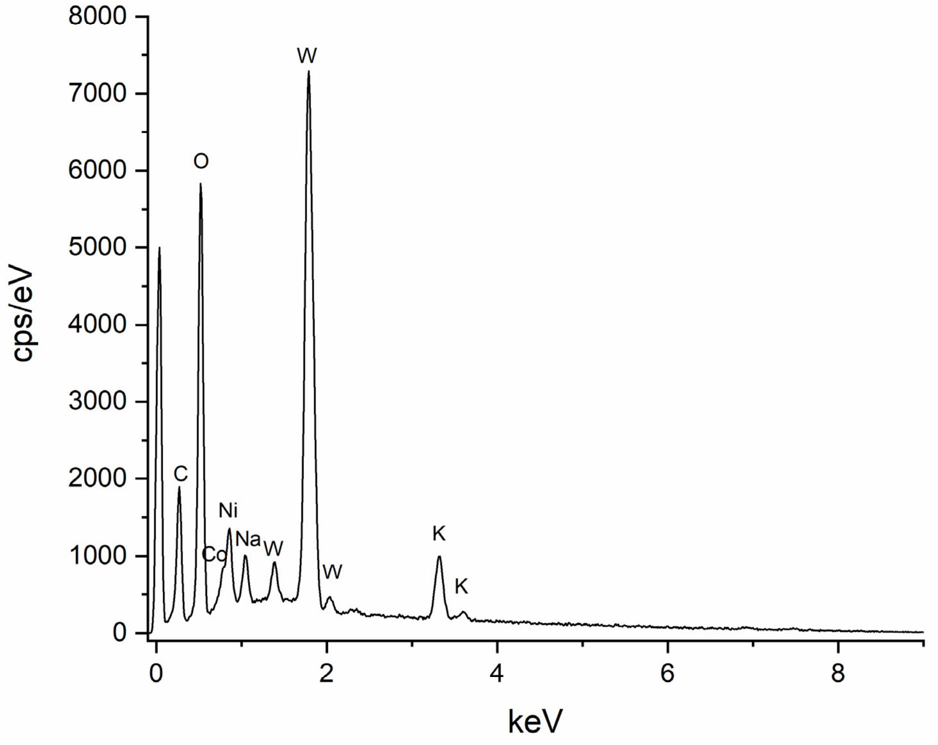

Figure S20. EDX spectrum of **CoNi(2)**.

Table S3. EDX analysis of **CoNi(2)**

**Element**	**Weight-%**	**Atomic-%**
C	9.03	28.07
O	20.09	46.89
Na	1.45	2.35
Si	0.33	0.44
K	6.28	5.99
Co	3.31	2.09
Ni	4.79	3.05
W	54.74	11.12

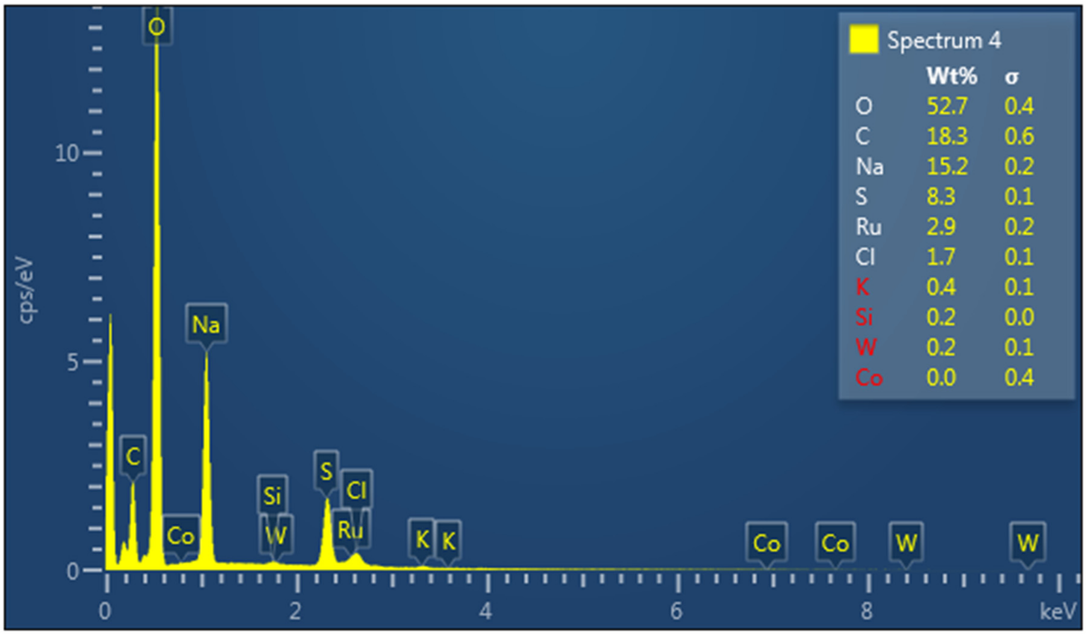

Figure S21. EDX measurement of lyophilized solution after Clark electrode tests of **Co(1)**.

Table S4. EDX analysis of **Co(1)**-POM-PS complex.

Element	Weight-%	Atomic-%
C	27.2	54.51
N	5.79	9.94
O	18.9	28.44
Co	0.5	0.21
Ru	5.46	1.30
W	42.02	5.50

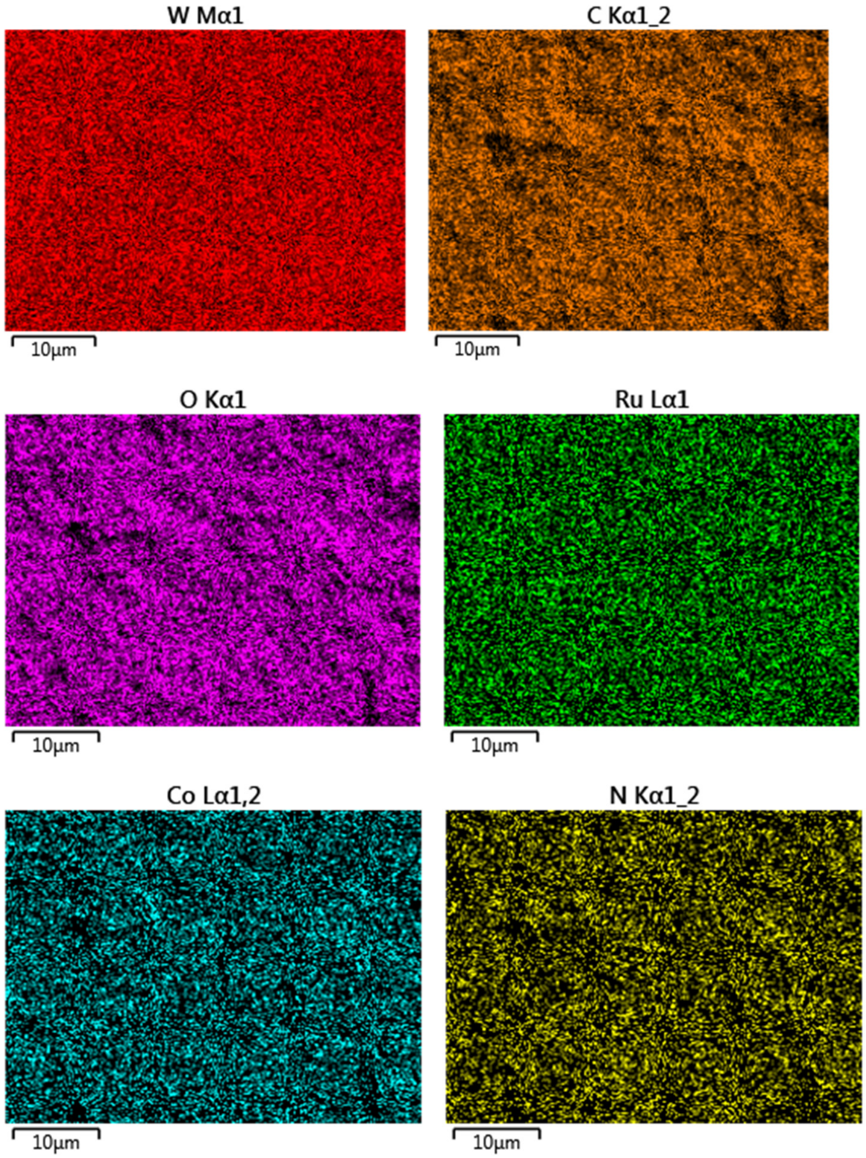

Figure S22. EDX-mapping of **Co(1)**-POM-PS complex.

Table S5. EDX analysis of **CoW300**.

Element	Weight-%	Atomic-%
O	21.78	64.70
Si	0.30	0.50
Co	10.29	8.30
W	59.12	15.28

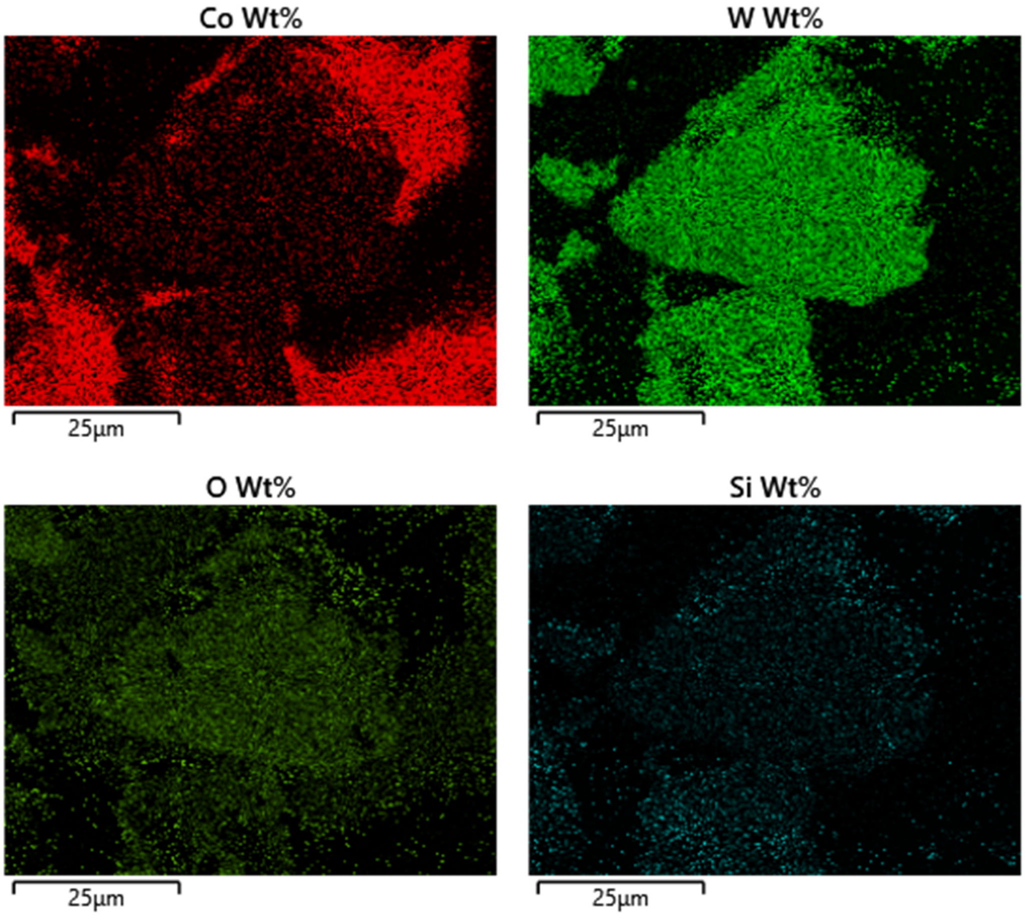

Figure S23. EDX mapping of **CoW300**.

Table S6. EDX analysis of **CoW400**.

Element	Weight-%	Atomic-%
O	16.89	38.74
Co	10.91	6.79
W	54.29	10.84

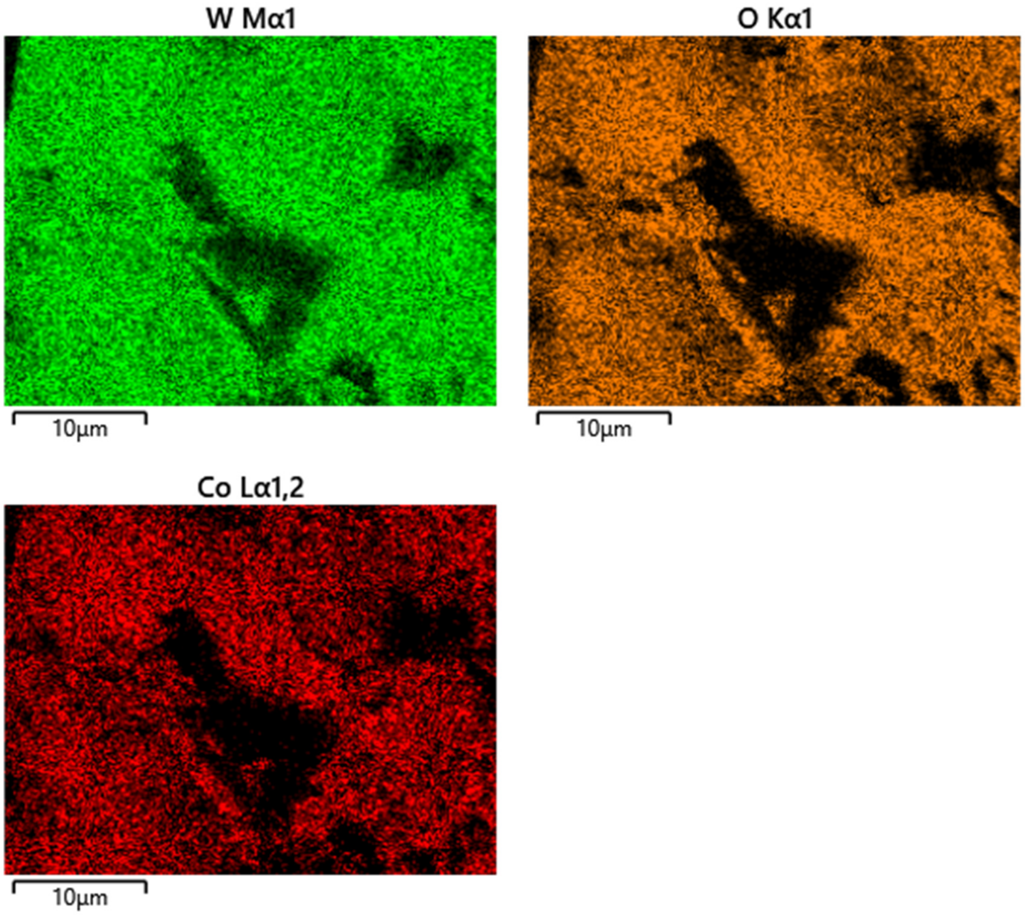

Figure S24. EDX mapping of **CoW400**.

Table S7. EDX analysis of **CoW500**.

Element	Weight-%	Atomic-%
O	18.62	60.26
Co	16.64	14.63
W	59.09	16.65

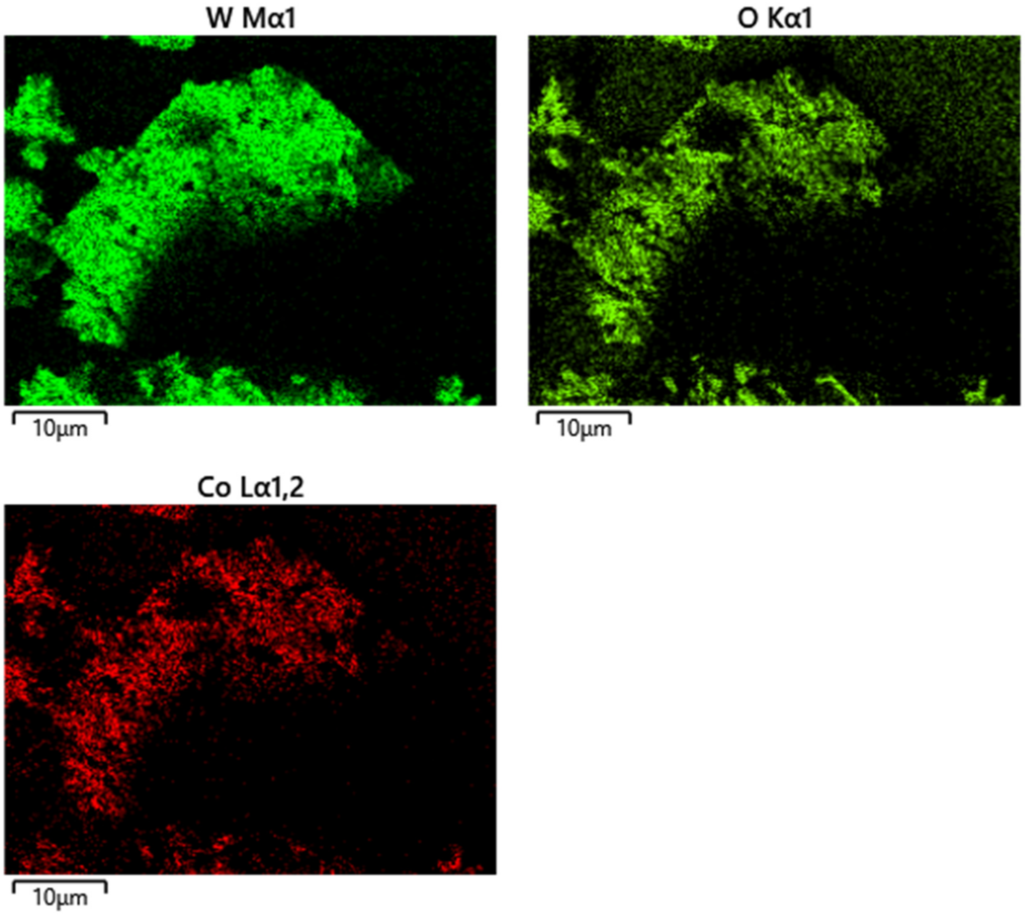

Figure S25. EDX mapping of **CoW500**.

Table S8. EDX analysis of **CoNi300**.

Element	Weight-%	Atomic-%
O	13.41	39.53
Co	5.44	4.36
Ni	8.78	7.05
W	57.89	14.85

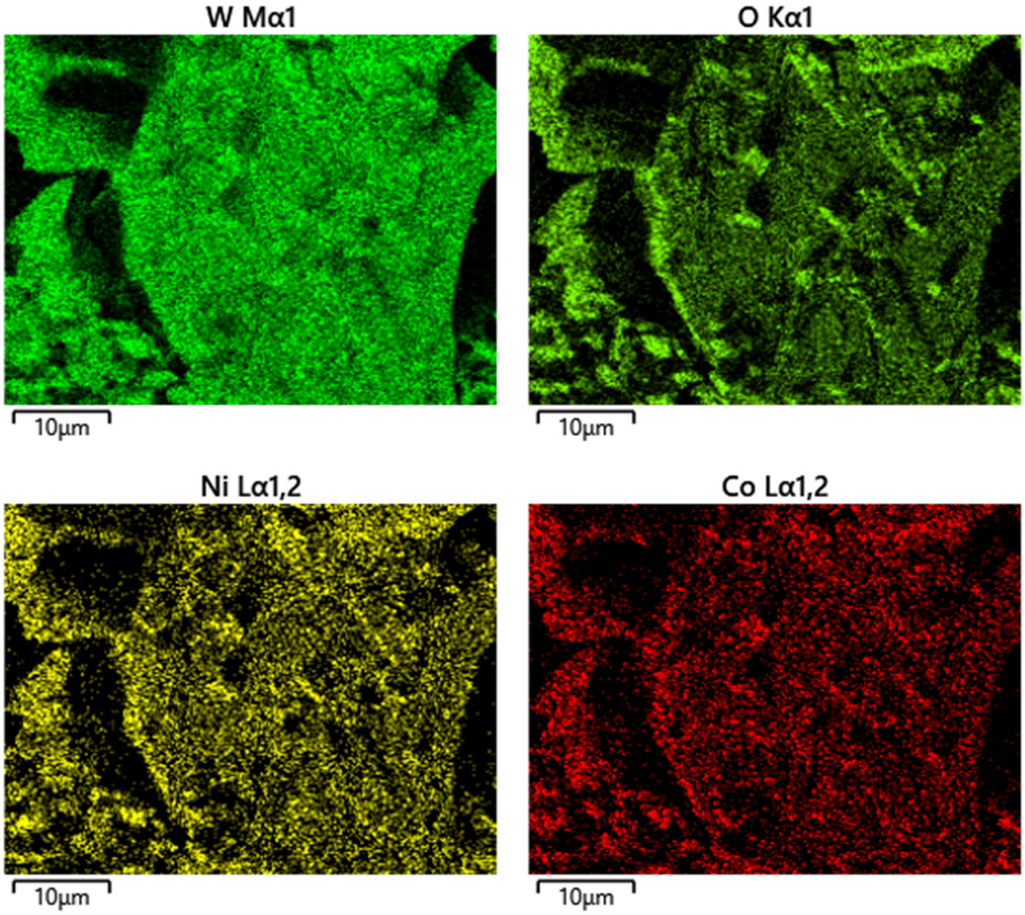

Figure S26. EDX mapping of **CoNi300**.

Table S9. EDX analysis of **CoNi400**.

Element	Weight-%	Atomic-%
O	23.19	64.62
Co	3.64	2.76
Ni	8.22	6.24
W	55.19	13.38

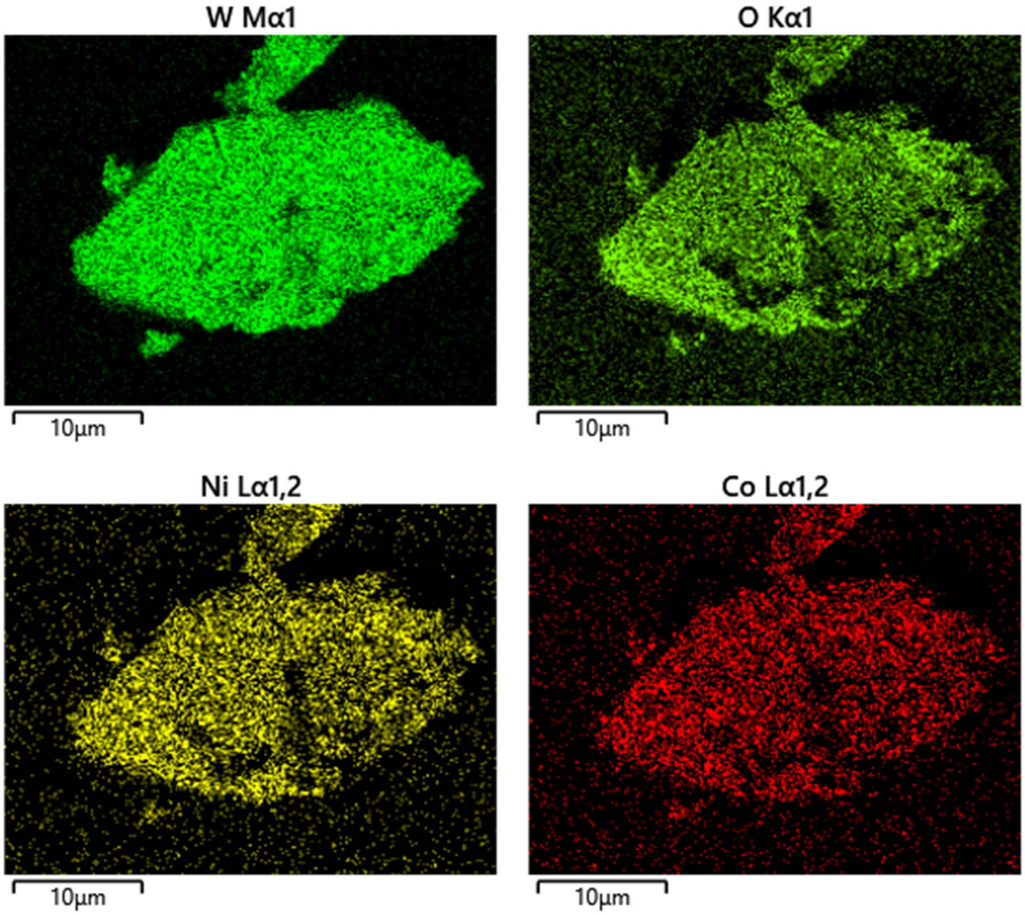

Figure S27. EDX mapping of **CoNi400**.

Table S10. EDX analysis of **CoNi500**.

Element	Weight-%	Atomic-%
O	24.42	65.20
Co	0.76	0.55
Ni	3.12	2.27
W	55.81	12.97

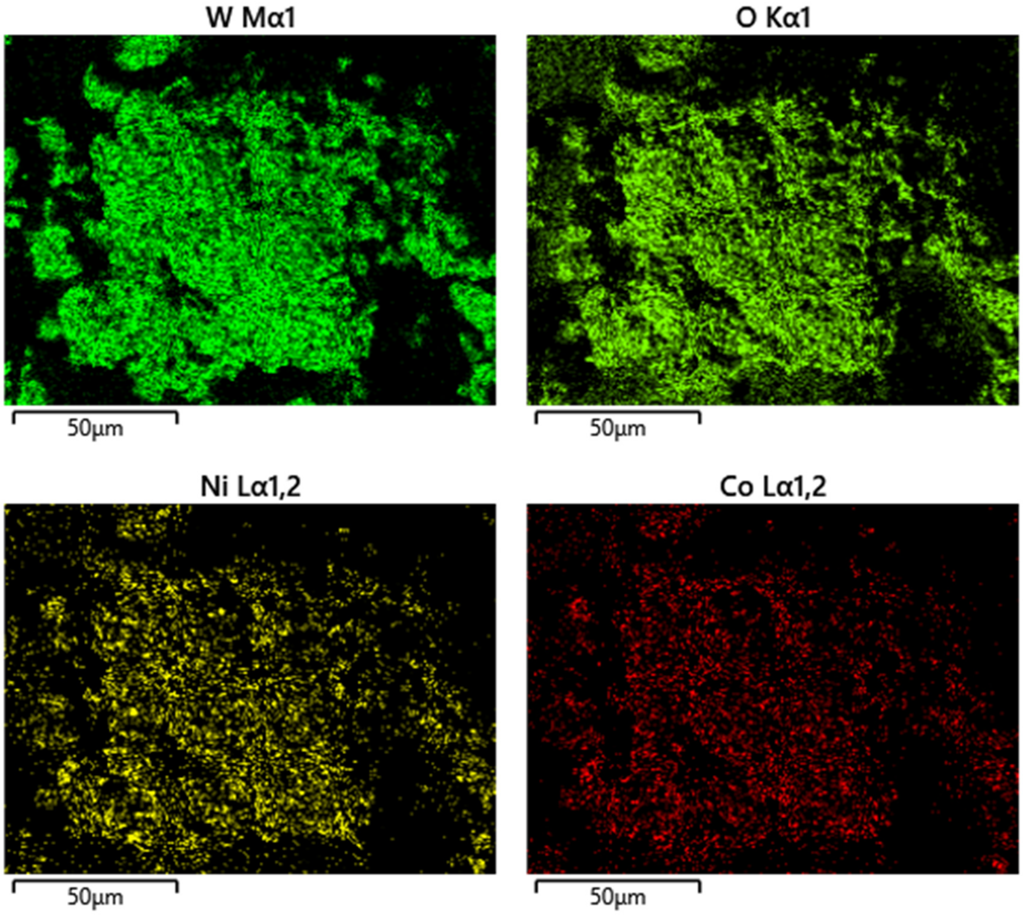

Figure S28. EDX mapping of **CoNi500**.

Table S11. ICP-MS analyses of pristine and calcination products of POM precursors.

**CoNi(2)**	Weight-%	Atomic-%	Co/Ni Ratio	Ratio Co/Ni : Si
**Co**	2.55	4.33	1.47	4.17 : 1
**Ni**	4.35	7.41	2.53	
**Si**	0.79	2.81		
**W**	48.1	26.16		

**CoW300**	Weight-%	Atomic-%
**Co**	9.14	15.57
**Si**	1.02	3.63
**W**	55	29.92

**CoW500**	Weight-%	Atomic-%
**Co**	8.92	15.20
**Si**	0.95	3.38
**W**	56.5	30.73

**CoNi300**	Weight-%	Atomic-%	Ratio Co/Ni
**Co**	2.85	4.86	0.48
**Si**	0.97	3.45	
**W**	54.1	29.43	
**Ni**	5.91	10.07	

Table S12. XPS analysis of **CoNi(2)**: normalized atomic concentration (at %) of all elements detected on a representative **CoNi(2)** sample.

at %	Co	Ni	W	O	C	K	Na
**CoNi(2)**	3.7	4.4	12.7	54.8	13.2	8.8	2.4

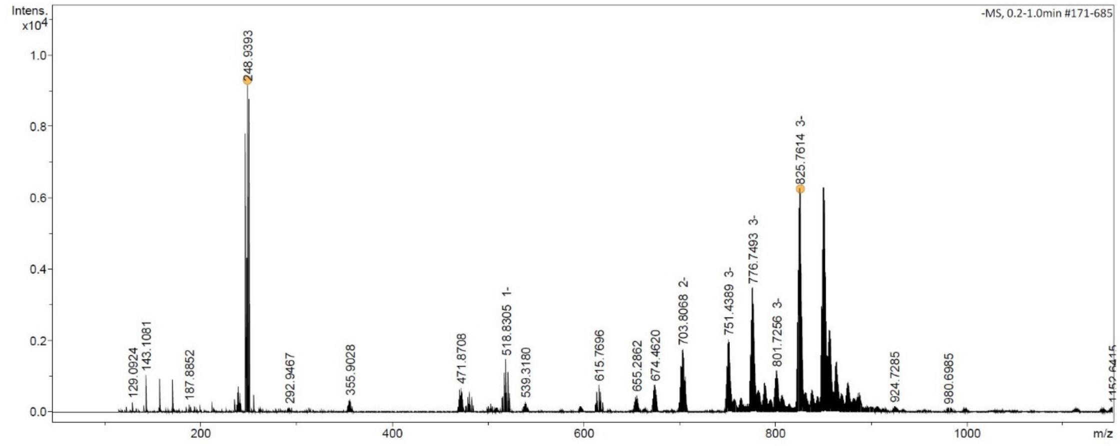

Figure S29. (-)-HR-ESI-MS of **Co(1)-**POM.

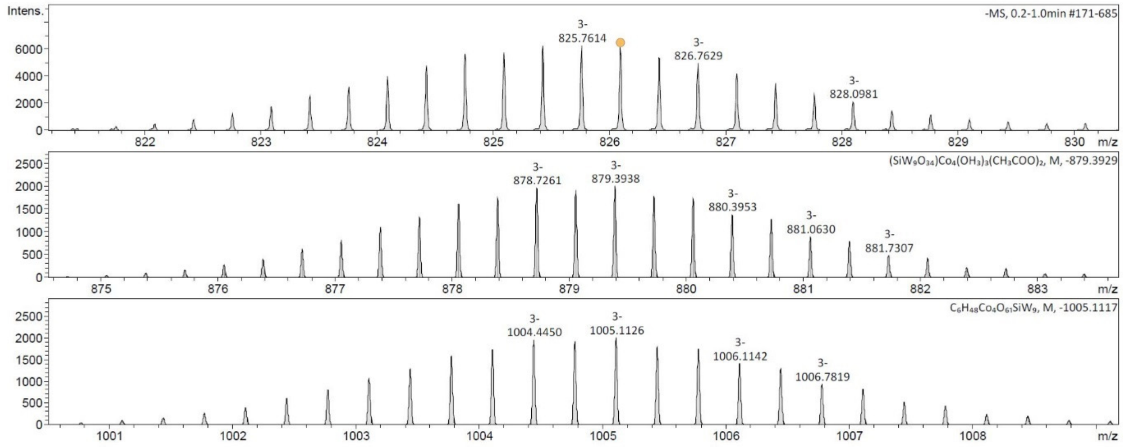

Figure S30. (-)-HR-ESI-MS of **Co(1)**-POM.

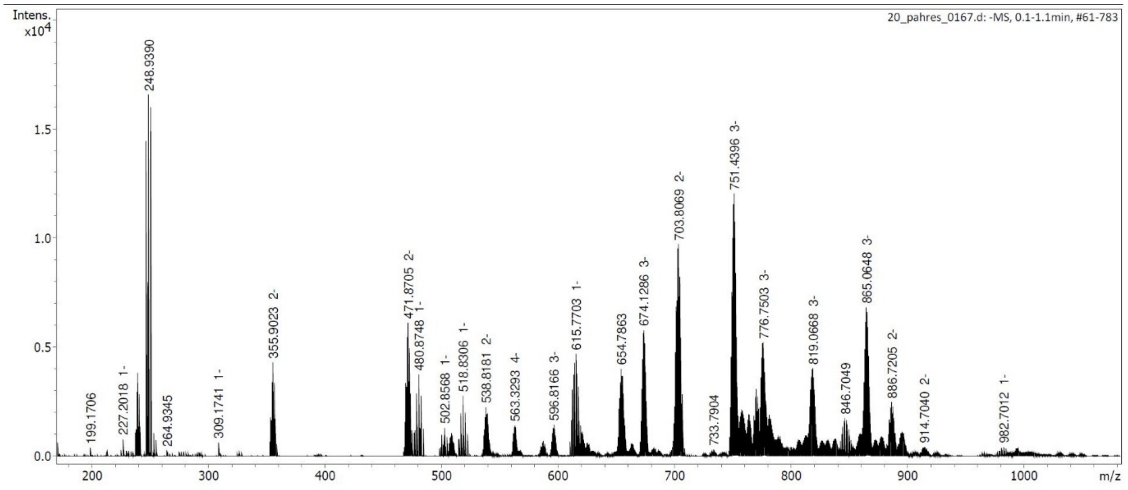

Figure S31. (-)-HR-ESI-MS of **CoNi(2)**.

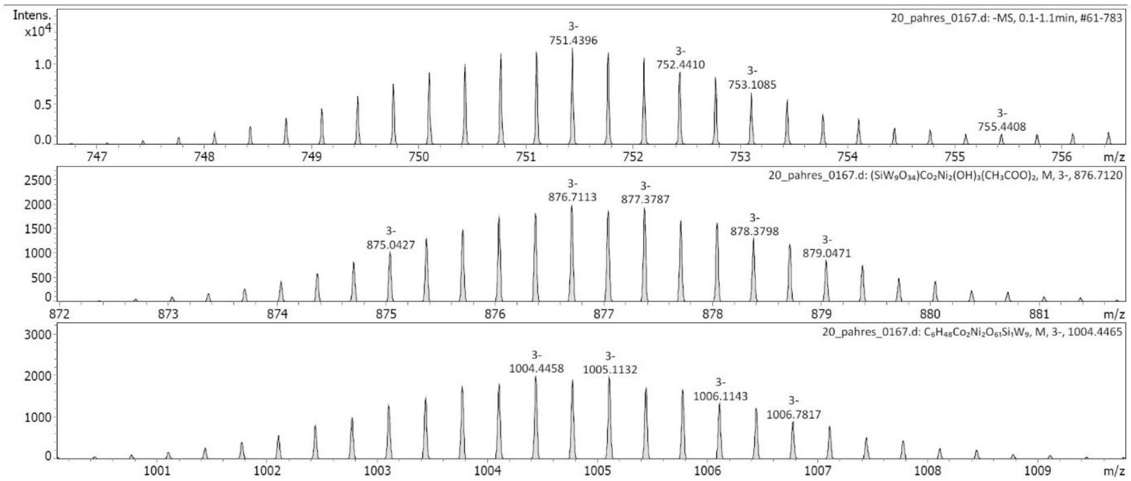

Figure S32. (-)-HR-ESI-MS of **CoNi(2)**.

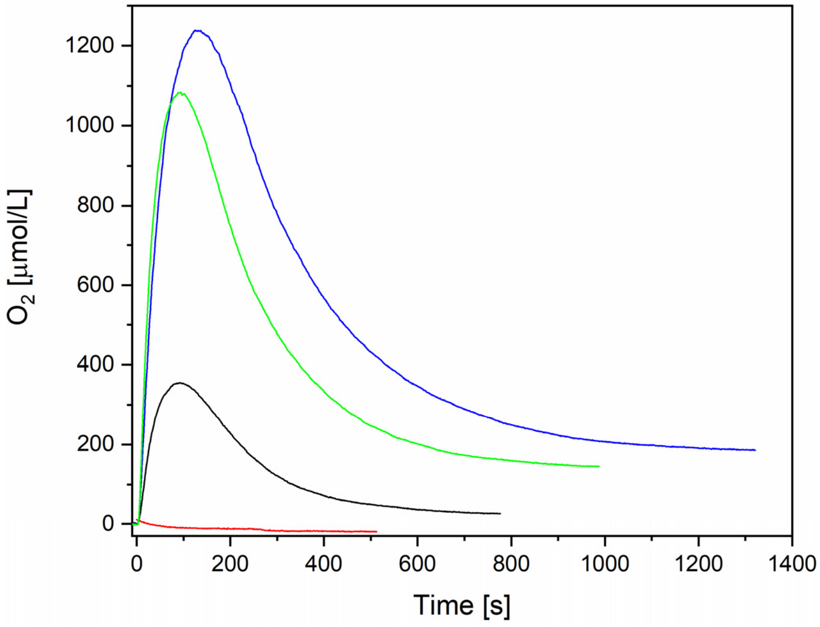

Figure S33. Clark-electrode kinetics of visible-light-driven O_2 evolution with **Co(1)** (20 µM) in different buffer conditions: acetate buffer (pH 4.75, red), sodium phosphate buffer (pH 7, black), borate buffer (pH 9, green) and borate buffer (pH 8, blue) with 1 mM $[Ru(bpy)_3]Cl_2$, 5 mM $Na_2S_2O_8$.

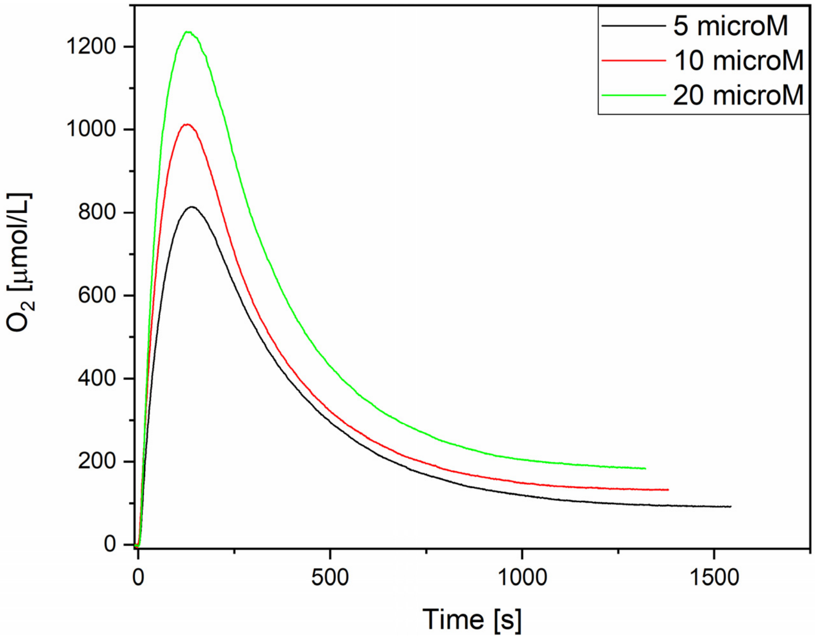

Figure S34. Clark-electrode kinetics of visible-light-driven O_2 evolution with different concentrations of **Co(1)**: 5 µM (black), 10 µM (red) and 20 µM (green), with 1 mM $[Ru(bpy)_3]Cl_2$, 5 mM $Na_2S_2O_8$ in 0.1 M borate buffer pH 8.

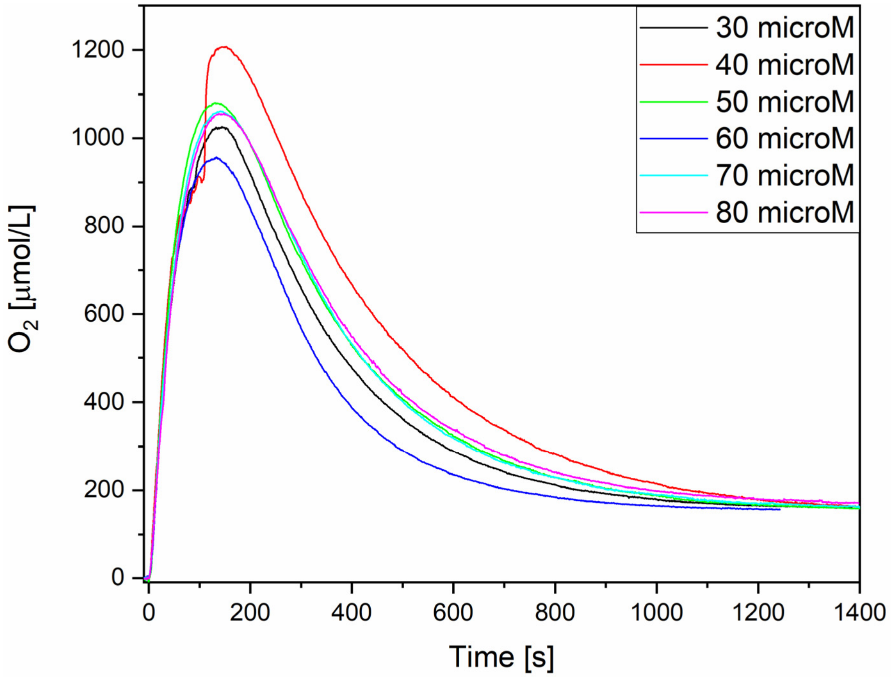

Figure S35. Clark-electrode kinetics of visible-light-driven O_2 evolution for different concentrations of **Co(1)**: 30 µM (black), 40 µM (red), 50 µM (green), 60 µM (dark blue), 70 µM (light blue), 80 µM (magenta) and 100 µM (yellow) with 1 mM [Ru(bpy)$_3$]Cl$_2$, 5 mM $Na_2S_2O_8$ in 0.1 M borate buffer pH 8.

Table S13. Catalytic activity of **Co(1)** over the concentration range 0 – 100 µM.

**WOC 1 [µmol/L]**	[a]**yield [%]**	[b]**TOF [s^{-1}]**	**TON**
0	4.18	-	-
1	9.44	0.5	235.4
5	26.35	0.5	131.4
10	38.60	0.5	96.3
20	47.96	0.5	59.8
**40**	**63.35**	**0.5**	**39.5**
50	56.84	0.5	28.4
60	54.87	0.5	22.8
80	55.61	0.5	17.3
100	64.05	0.5	16.0

[a]persulfate yield is based on a theoretical O_2 yield corresponding to 50% of the initial $Na_2S_2O_8$ amount; [b]TOF values were determined as follows: initial linear slope of O_2 evolution divided by time and catalyst concentration.

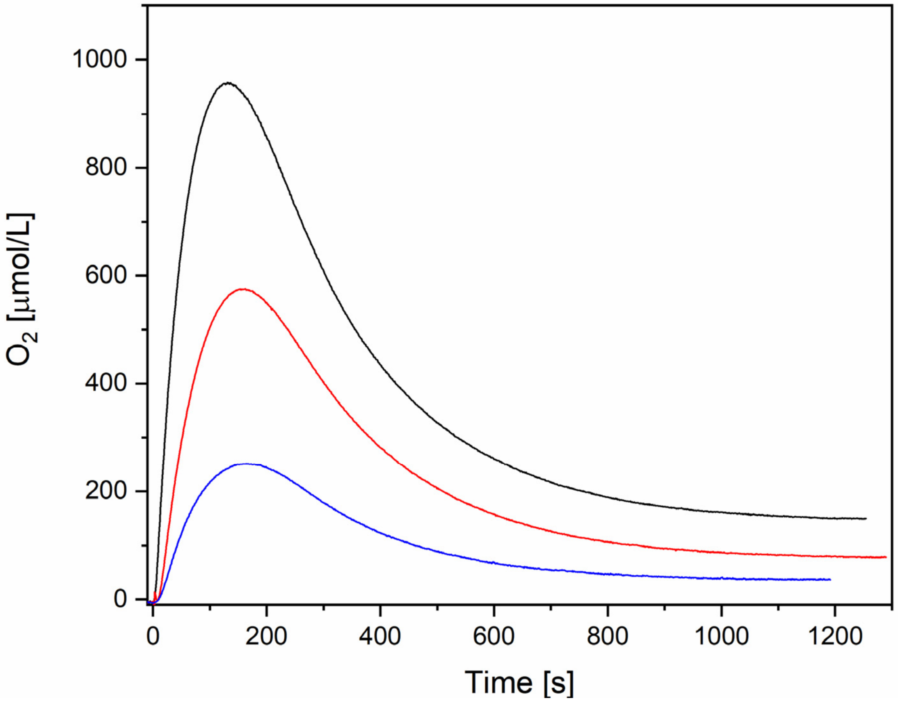

Figure S36. Clark-electrode kinetics of visible-light-driven O_2 evolution recycling test with 40 µM **Co(1)**, 1 mM $[Ru(bpy)_3]Cl_2$, 5 mM $Na_2S_2O_8$ in 0.1 M borate buffer pH 8: 1st cycle (black), 2nd cycle (red), 3rd cycle (blue).

The 2nd recycling test (red curve) was performed by adding 5 mM of fresh $Na_2S_2O_8$ to the solution after readjusting the pH of the post-catalytic mixture to 8. The 3rd recycling test (blue curve) started from adjustment of the 1st recycled solution to pH 8 and addition of 5 mM $Na_2S_2O_8$.

Table S14. Catalytic activity of **Co(1)** in three recycling tests (40 µM WOC).

^{a}**test**	^{b} **O_2 yield**	^{c}**TOF [s^{-1}]**	**TON**
1st cycle	56.35	0.5	35.1
2nd cycle	25.31	0.2	15.8
3rd cycle	10.97	0.1	6.84

^{a}Experiments were performed at pH 8 in 0.1 M borate buffer; ^{b}persulfate yield is based on a theoretical O_2 yield corresponding to 50% of the initial $Na_2S_2O_8$ amount; ^{c}TOF values were determined as follows: initial linear slope of O_2 evolution divided by time and catalyst concentration.

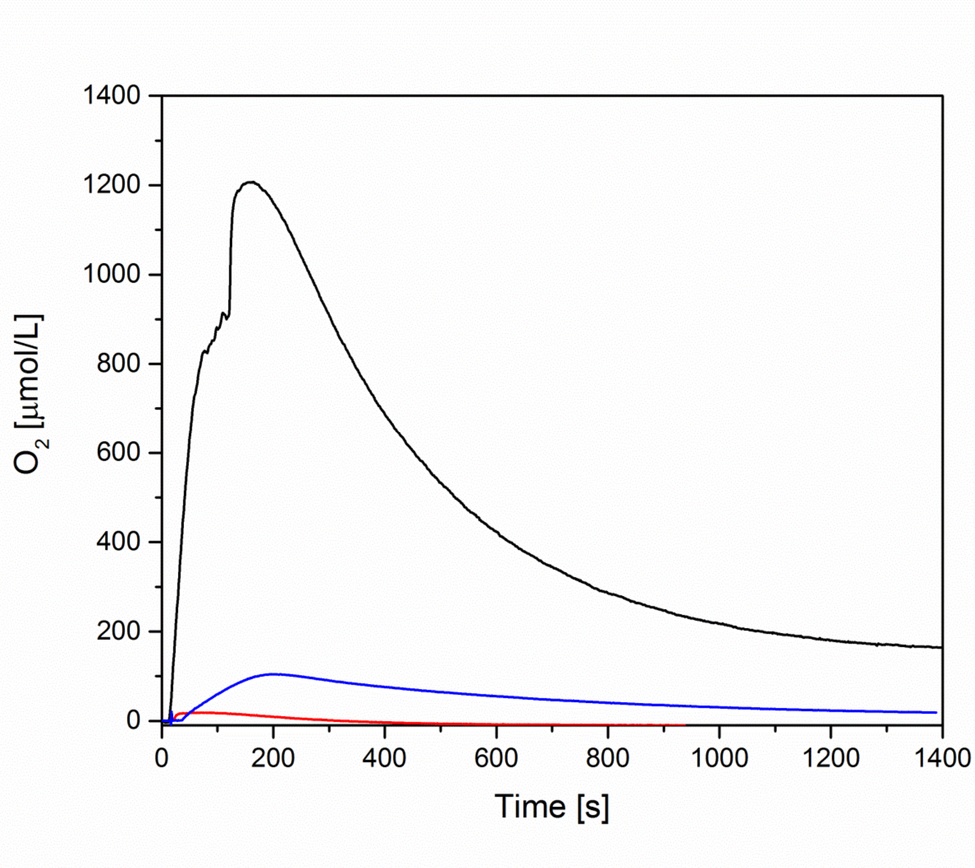

Figure S37. Clark-electrode kinetics of visible-light-driven O_2 evolution filter test with 40 µM **Co(1)**, 1 mM $[Ru(bpy)_3]Cl_2$, 5 mM $Na_2S_2O_8$ in 0.1 M borate buffer pH 8. 1st cycle (black), filtered solution with 5 mM $Na_2S_2O_8$ added (red), and with 1 mM $[Ru(bpy)_3]Cl_2$, 5 mM $Na_2S_2O_8$ added in 0.1 M borate buffer pH 8 (blue).

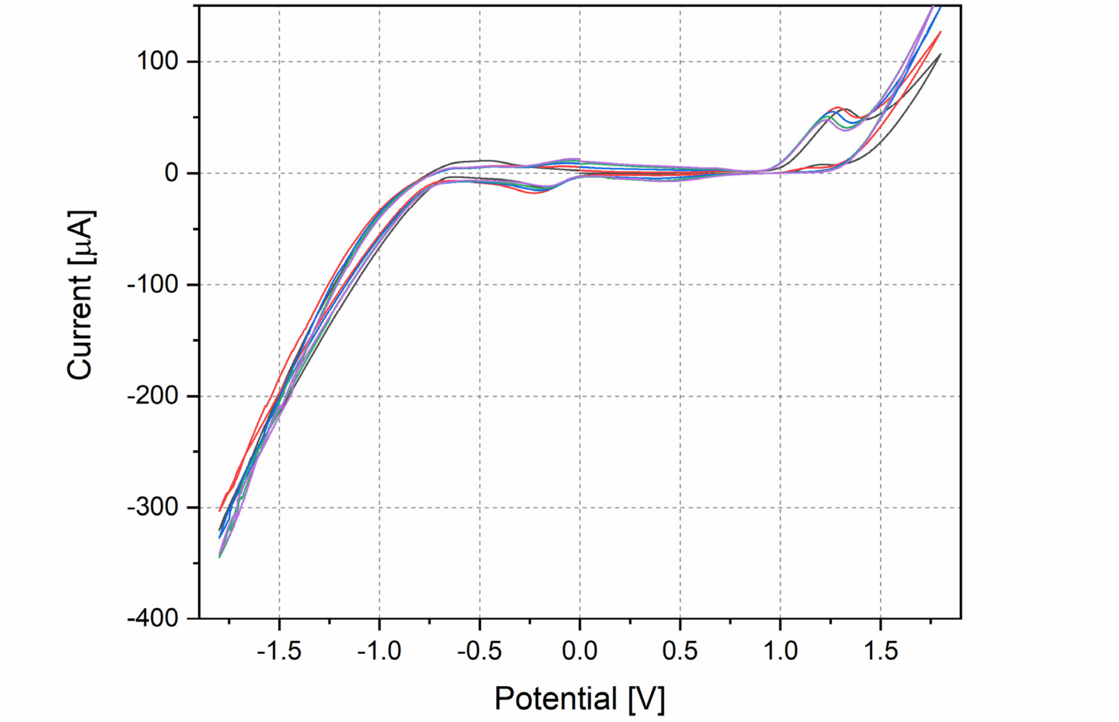

Figure S38. Cyclic voltammogram of 50 μM **Co(1)** in 0.1 M borate buffer (0.1 M, pH 8; scan rate: 20 mV/s).

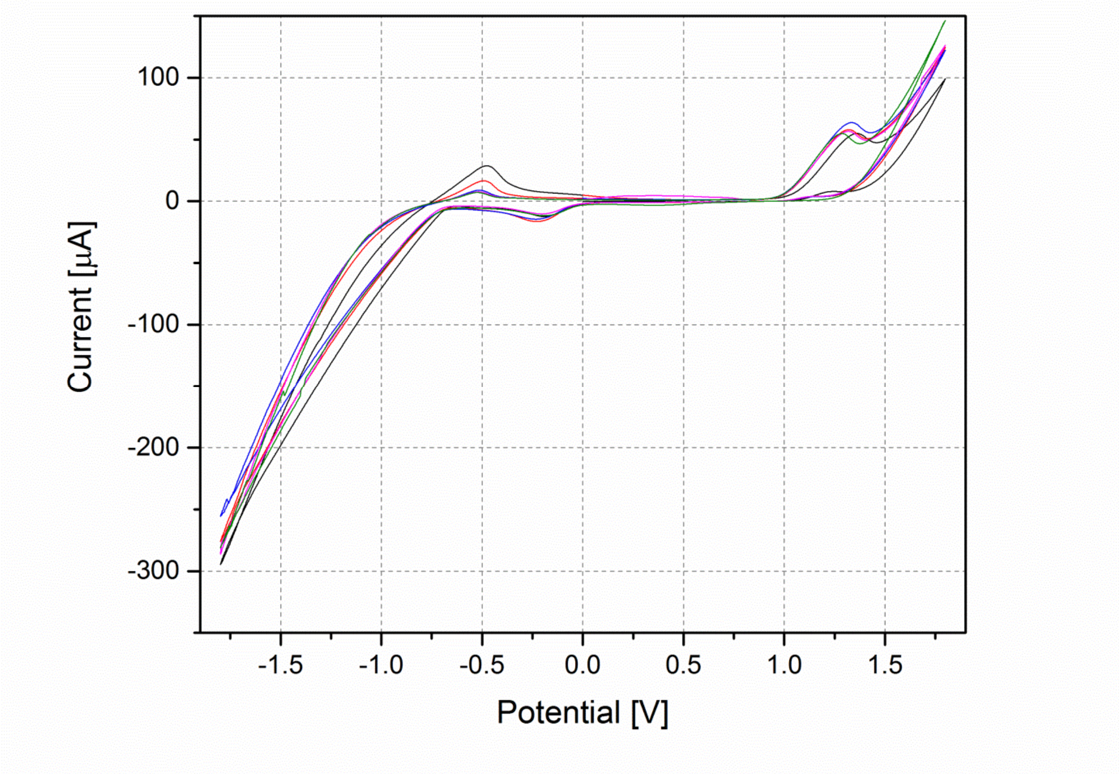

Figure S39. Cyclic voltammogram of 50 μM **CoNi(2)** in 0.1 M borate buffer (0.1 M, pH 8; scan rate: 20 mV/s).

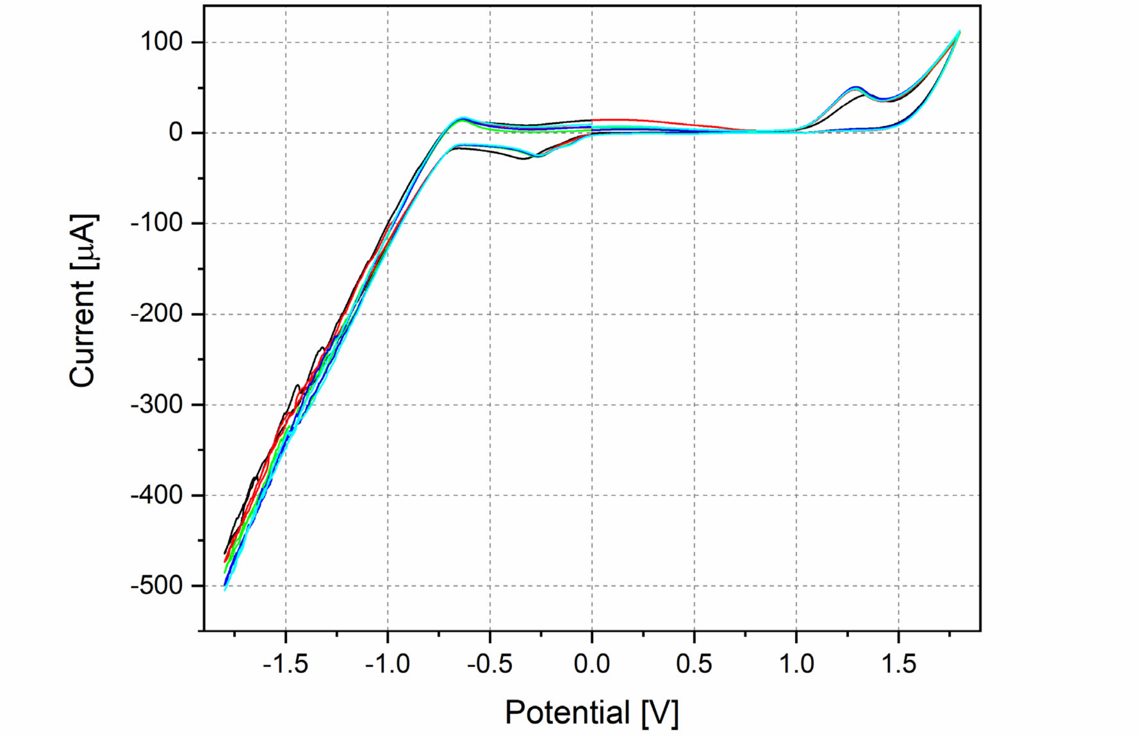

Figure S40. Cyclic voltammogram of 50 μM **Co(1)**, 1 mM $[Ru(bpy)_3]Cl_2$ and 5 mM $Na_2S_2O_8$ in 0.1 M borate buffer pH 8 (scan rate: 20 mV/s).

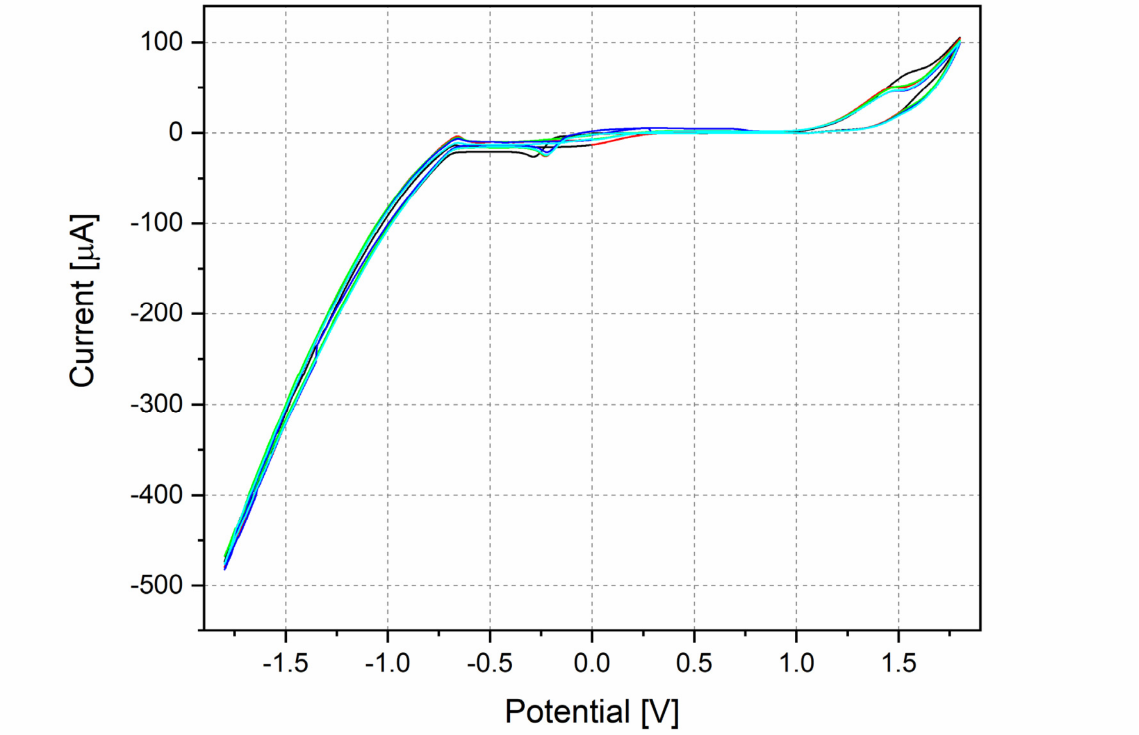

Figure S41. Cyclic voltammogram of 1 mM $[Ru(bpy)_3]Cl_2$ and 5 mM $Na_2S_2O_8$ in 0.1 M borate buffer pH 8 (scan rate: 20 mV/s).

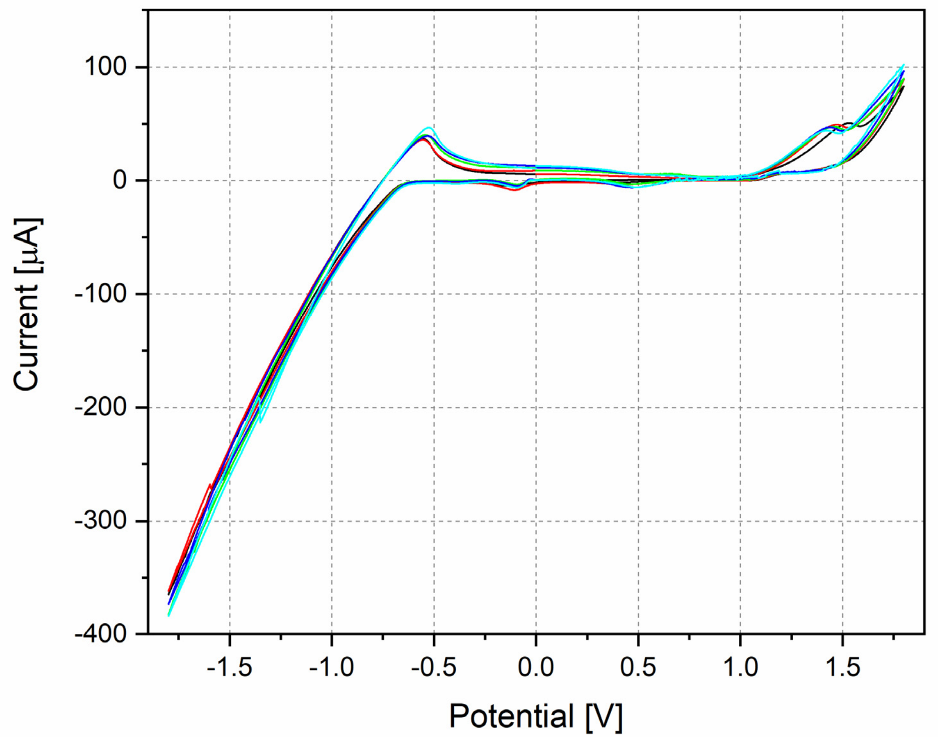

Figure S42. Cyclic voltammogram of 1 mM $[Ru(bpy)_3]Cl_2$ in 0.1 M borate buffer pH 8 (scan rate: 20 mV/s).

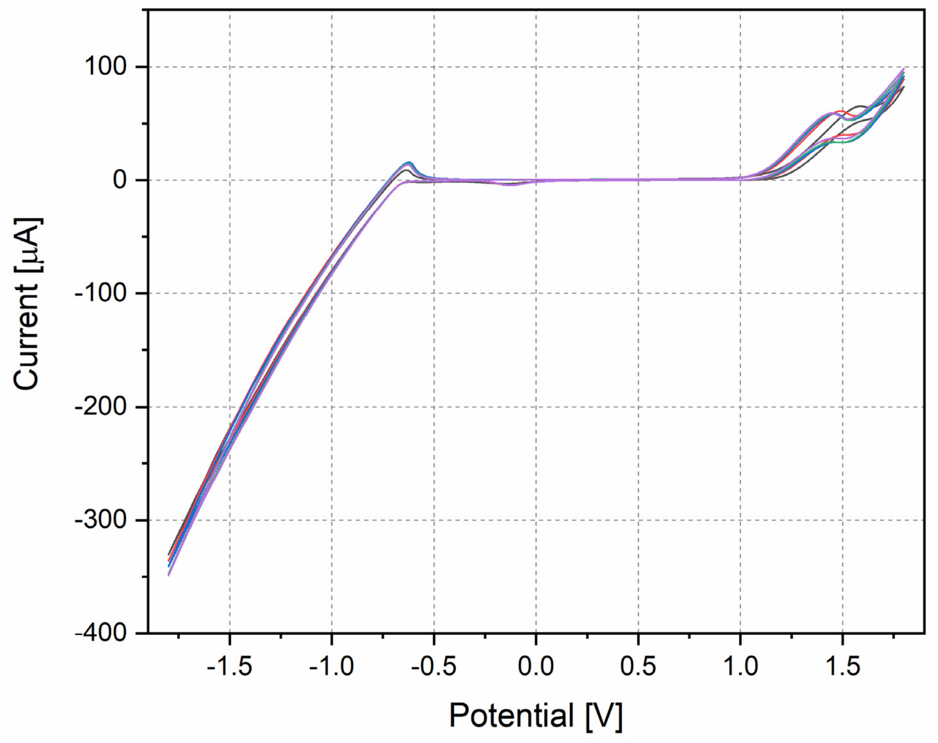

Figure S43. Cyclic voltammogram of 0.1 M borate buffer pH 8 (scan rate: 20 mV/s).

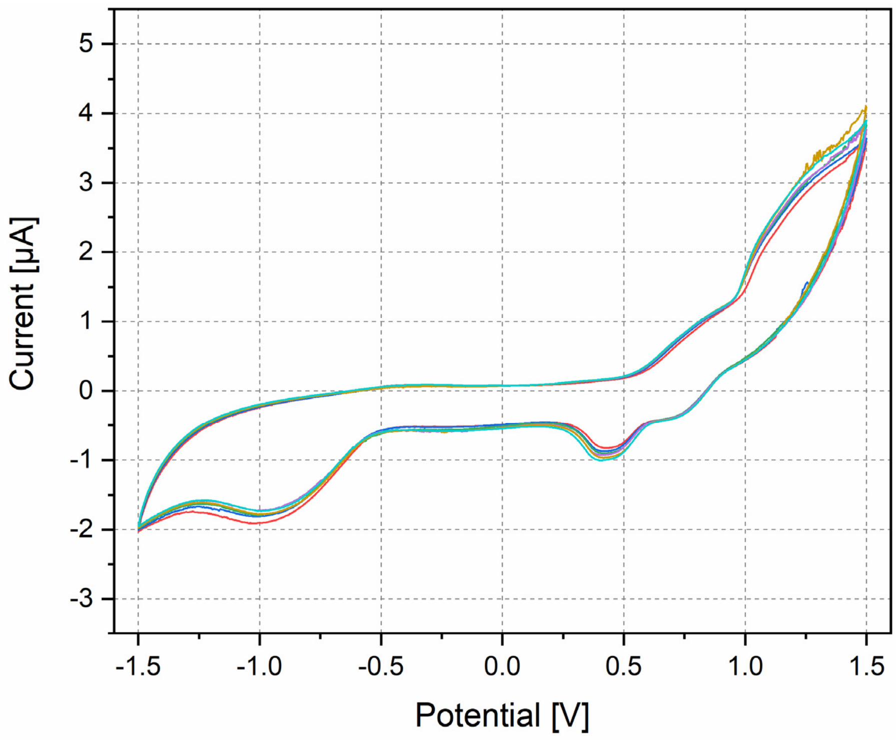

Figure S44. Cyclic voltammogram of **Co(1)**-POM-PS complex in 0.1 M borate buffer pH 8.

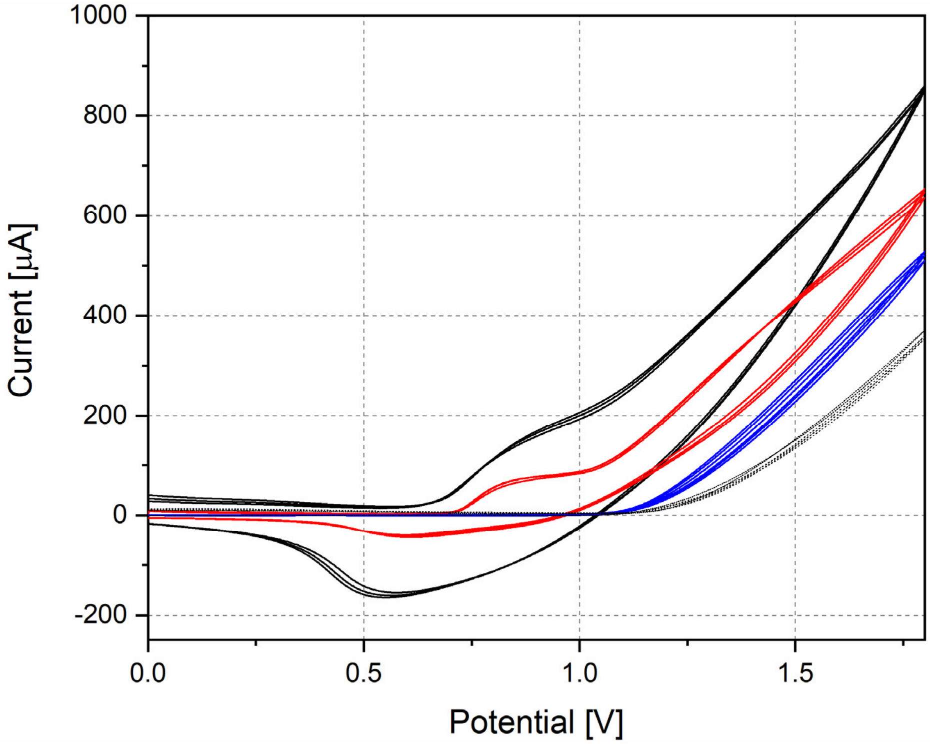

Figure S45. Cyclic voltammogram of **CoW300** (black), **CoW400** (red), **CoW500** (blue) and FTO plate (dotted line; scan rate: 20 mV/s).

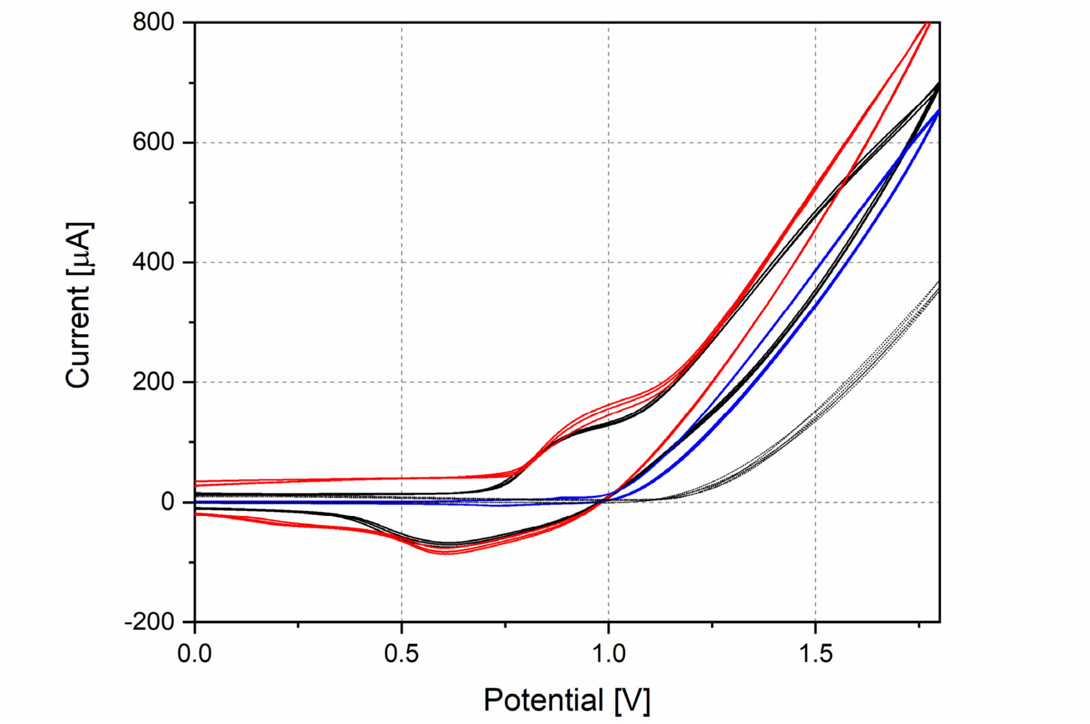

Figure S46. Cyclic voltammogram of **CoNi300** (black), **CoNi400** (red), **CoNi500** (blue) and FTO plate (dotted line; scan rate: 20 mV/s).

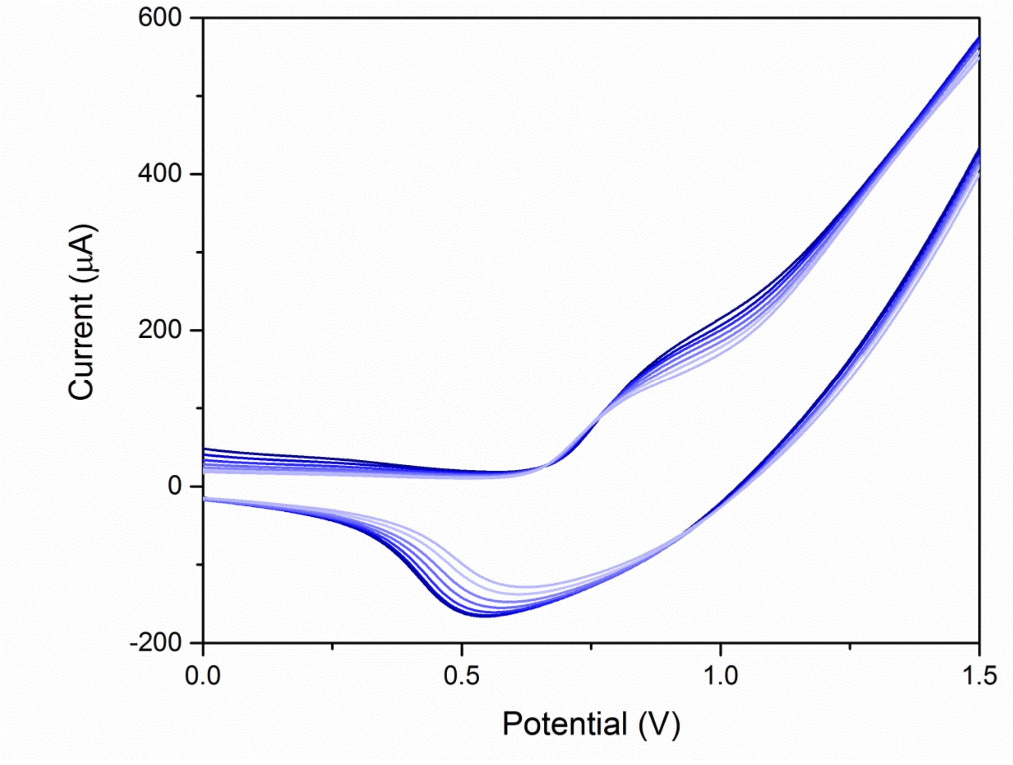

Figure S47. Cyclic voltammogram of **CoW300** (scan rate: 20 mV/s).

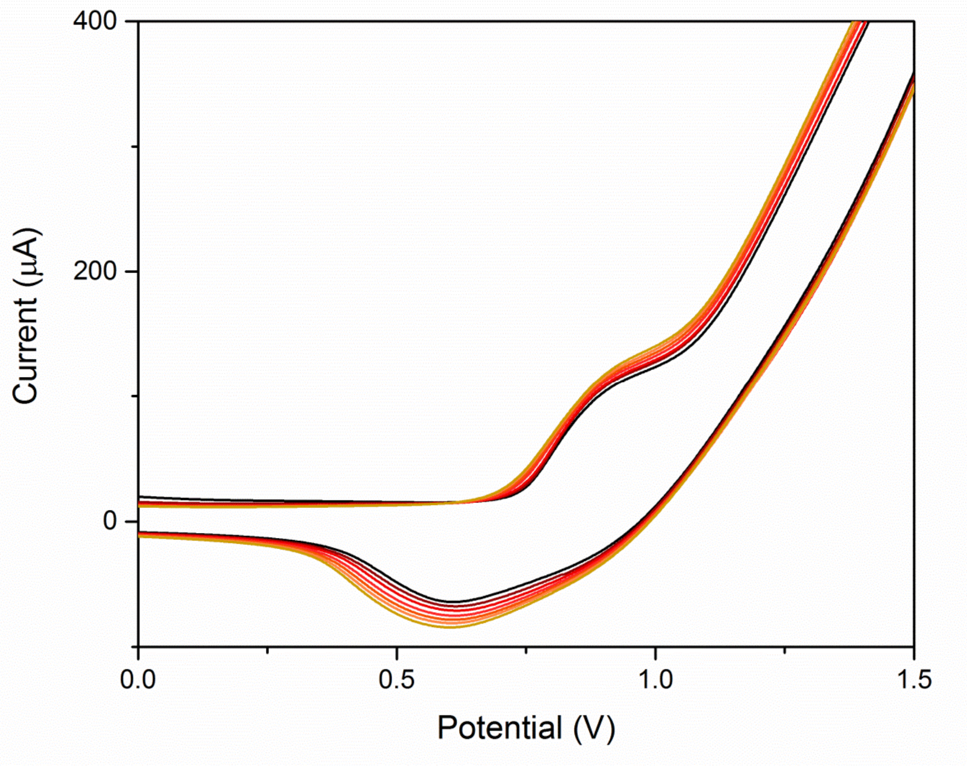

Figure S48. Cyclic voltammogram of **CoNi300** (scan rate: 20 mV/s).

References

[1] Rigaku Oxford Diffraction, 2015, **31**.

[2] R. C. Clark, J. S. Reid, *Acta Cryst. A* 1995, **51**, 887.

[3] *CrysAlisPro* (version 1.171.40.39a), Rigaku Oxford Diffraction, **2018**.

[4] Dolomanov, O. V.; Bourhis, L. J.; Gildea, R. J.; Howard, J. A. K.; Puschmann, H. *J. Appl. Cryst.,* 2009, **42**, 339.

[5] Sheldrick, G. M. *Acta Cryst. A,* 2015, **71**, 3.

[6] Sheldrick, G. M. *Acta Cryst. C,* 2015, **71**, 3.

[7] Spek, A. L. *Acta Cryst. D,* 2009, **65**, 148.

[8] H. Jia, J. Stark, L. Q. Zhou, C. Ling, T. Sekito, Z. Markin, *RSC Adv.,* 2012, **2**, 10874-10881.
